# The CMG (CDC45/RecJ, MCM, GINS) complex is a conserved component of the DNA replication system in all archaea and eukaryotes

**DOI:** 10.1186/1745-6150-7-7

**Published:** 2012-02-13

**Authors:** Kira S Makarova, Eugene V Koonin, Zvi Kelman

**Affiliations:** 1 National Center for Biotechnology Information, NLM, National Institutes of Health, Bethesda, Maryland 20894, USA; 2 National Institute of Standards and Technology, Institute for Bioscience and Biotechnology Research, University of Maryland, 9600 Gudelsky Drive, Rockville, MD 20850, USA

## Abstract

**Background:**

In eukaryotes, the CMG (CDC45, MCM, GINS) complex containing the replicative helicase MCM is a key player in DNA replication. Archaeal homologs of the eukaryotic MCM and GINS proteins have been identified but until recently no homolog of the CDC45 protein was known. Two recent developments, namely the discovery of archaeal GINS-associated nuclease (GAN) that belongs to the RecJ family of the DHH hydrolase superfamily and the demonstration of homology between the DHH domains of CDC45 and RecJ, show that at least some Archaea possess a full complement of homologs of the CMG complex subunits. Here we present the results of in-depth phylogenomic analysis of RecJ homologs in archaea.

**Results:**

We confirm and extend the recent hypothesis that CDC45 is the eukaryotic ortholog of the bacterial and archaeal RecJ family nucleases. At least one RecJ homolog was identified in all sequenced archaeal genomes, with the single exception of *Caldivirga maquilingensis*. These proteins include previously unnoticed remote RecJ homologs with inactivated DHH domain in *Thermoproteales*. Combined with phylogenetic tree reconstruction of diverse eukaryotic, archaeal and bacterial DHH subfamilies, this analysis yields a complex scenario of RecJ family evolution in Archaea which includes independent inactivation of the nuclease domain in Crenarchaeota and Halobacteria, and loss of this domain in Methanococcales.

**Conclusions:**

The archaeal complex of a CDC45/RecJ homolog, MCM and GINS is homologous and most likely functionally analogous to the eukaryotic CMG complex, and appears to be a key component of the DNA replication machinery in all Archaea. It is inferred that the last common archaeo-eukaryotic ancestor encoded a CMG complex that contained an active nuclease of the RecJ family. The inactivated RecJ homologs in several archaeal lineages most likely are dedicated structural components of replication complexes.

**Reviewers:**

This article was reviewed by Prof. Patrick Forterre, Dr. Stephen John Aves (nominated by Dr. Purificacion Lopez-Garcia) and Prof. Martijn Huynen.

For the full reviews, see the Reviewers' Comments section.

## Background

### Replication complexes in Archaea and Eukarya

The eukaryotic minichromosome maintenance (MCM) complex consists of six paralogous proteins (MCM2-7) which belong to a distinct family within the AAA+ superfamily of ATPases. All MCM complex subunits are essential for cell viability and are required for the initiation of DNA replication and replication fork progression. Genetic, biochemical and structural studies have shown that the MCM complex is the replicative helicase that is responsible for the separation of the DNA strands during chromosomal replication [1,2]. However, *in vitro *and *in vivo *experiments have demonstrated that the MCM complex, on its own, is not the active helicase but requires the association with two accessory factors, the tetrameric GINS complex (Sld5, Psf1-3) and the CDC45 protein. This complex is referred to as the CMG (CDC45, MCM, GINS) complex and is thought to be the active replicative helicase unit *in vivo *[3-5]. In addition to binding to MCM, the GINS complex has also been shown to associate with Polα-primase, the protein complex that synthesizes the primers on the lagging strand, and with the leading and lagging strands polymerases, Polε the Polδ, respectively (reviewed in [6]).

Homologs of MCM are also thought to function as the replicative helicases in Archaea (summarized in [7-9]). Most archaeal genomes encode a single MCM homologue that forms homohexamers. In contrast to the eukaryotic MCM2-7 complex, which does not exhibit helicase activity without the associated GINS and CDC45 proteins, *in vitro *experiments with the homohexamers from several Archaea have shown that the archaeal enzymes possess robust helicase activity on their own ([9] and references therein). In several archaeal species, multiple MCM paralogs have been identified. To date, however, only one study has been published on the three MCM proteins from the archaeon *Thermococcus kodakarensis *[10]. Although this organism encodes three MCM paralogs, only one protein, MCM3 (encoded by the TK1620 gene), is essential for cell viability. MCM3 forms active homohexamers in solution and possesses biochemical properties similar to those of other MCM helicases.

The archaeal GINS complex follows the same evolutionary pattern as the MCM complex: it is also tetrameric, but unlike the eukaryotic complex, does not contain four different polypeptides. Instead, in most archaea the GINS complex contains two copies of a subunit homologous to eukaryotic Psf1 and Sld5 (this dimer is referred to as GINS15), and two copies of a subunit homologous to eukaryotic Psf2 and Psf3 (GINS23). Several archaea encode a single GINS homolog which forms a homotetramer. The structure of the heterotetrameric *T. kodakarensis *GINS is similar overall to the human complex although the contacts between the GINS15 and GINS23 subunits differ [11]. The main structural difference between the two is the location of the C-terminal domain of the archaeal GINS15 subunit, which is located about 30 Å away from the corresponding position of Psf1 subunits in the eukaryotic structures [11].

In *Sulfolobus solfataricus *the GINS complex is additionally associated with a protein called RecJdbd (RecJ-like DNA-binding domain) that is homologous to the C-terminal domain of bacterial RecJ but lacks a counterpart to the nuclease domain of RecJ [12]. The GINS complex of the euryarchaeon *T. kodakarensis *has been recently shown to interact with primase, MCM, DNA polymerase D, PCNA and the GINS-associated nuclease (GAN) [13,14]. Unlike the RecJdbd of *S. solfataricus *GAN is a *bona fide *ortholog of bacterial RecJ containing a clearly identifiable DHH phosphoesterase domain with all the essential catalytic residues. The biochemical properties of the GAN nuclease are also similar to those of the bacterial RecJ with respect to substrate requirements, metal co-factor, and directionality.

Recently, it has been shown using sensitive sequence analysis methods that the N-terminal region of CDC45 contains a DHH phosphoesterase domain leading to the hypothesis that CDC45 is the eukaryotic ortholog of RecJ [15,16]. Considering that Archaea typically possess homologs of the essential components of the eukaryotic replication machinery [17-19] and that CDC45 (the apparent RecJ ortholog) is essential for replication, it could be predicted that Archaea possess a conserved counterpart to the RecJ-MCM-GINS complex. In order to test this hypothesis and gain further insight into the evolution of the archaeal replication apparatus, we undertook an in-depth phylogenomic analysis of the DHH phosphoesterase superfamily that includes the RecJ family of nucleases.

## Results and Discussion

### Phylogenomics of the RecJ family

Inspection of the arCOG database [20], which consists of clusters of othologous genes from the sequenced archaeal genomes, reveals a complex distribution of the RecJ homologs. Many Euryarchaeota encode two or more paralogous proteins of arCOG00427 that appear to be orthologous to RecJ (the GAN nuclease, in particular, belongs in this group). In contrast, the majority of Crenarchaeota lack members of this arCOG; the aforementioned RecJdbd of *S. solfataricus *belongs to a distinct arCOG05902 that is specific to *Sulfolobales*.

**Figure 1:**
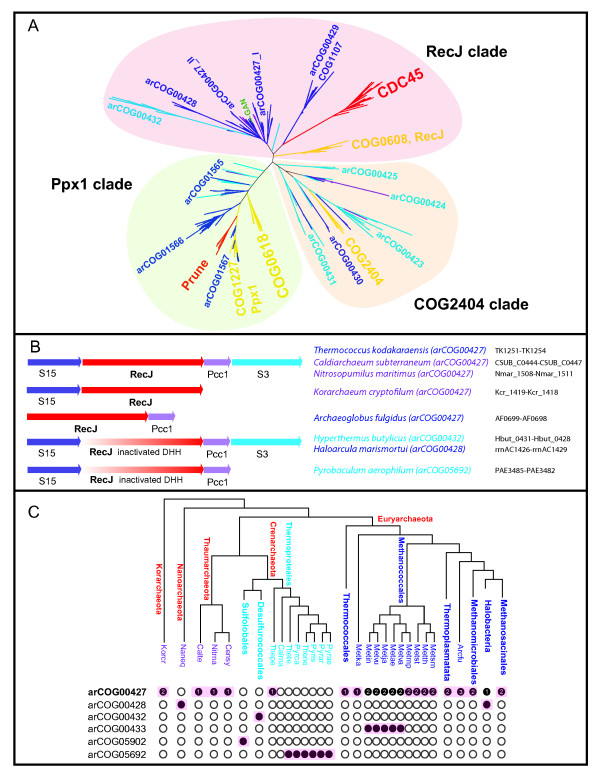
**RecJ homologs in archaea**. Color code: eukaryotes - red; bacteria - yellow; euryarchaea - dark blue; crenarchaea - light blue; deeply branched archaeal lineages (Thaumarchaeota, Korarchaeota, Nanoarchaeota) - purple. A. Phylogeny of the DHH superfamily, The tree was constructed using aligned blocks corresponding to DHH domain (229 sequences in total, 83 aligned positions; see Additional file 1 for details). arCOG or COG numbers or family name (for eukaryotes) are indicated for the corresponding branches. Three distinct major clades are shaded. The location of the GAN protein is indicated by green. B. Genomic context of the RecJ homologs in selected archaea, Orthologous genes are shown by arrows of the same color; genes shown approximately to scale. The arCOGs to which RecJ-like proteins are assigned are indicated in parenthesis. The protein IDs for this region in the corresponding genomes are provided. C. The phyletic patterns of RecJ-related arCOGs, The tree is a modified version of the consensus tree representing phylogeny of archaea [38] with *Caldiarchaeum subterraneum *included in the Thaumarchaeota branch and several branches with consistent distribution of RecJ-related subfamilies are collapsed. The phyletic patterns for indicated arCOGs (filled circles show presence and empty circles show absence) are superimposed over the phylogenetic tree of archaea. The circles for proteins implicated in replication are shaded. The number of paralogs is indicated inside the circles for arCOG00427 (all other subfamilies have one paralog for each genome). Abbreviations: Arcfu - *Archaeoglobus fulgidus*; Metsm - *Methanobrevibacter smithii *ATCC 35061; Metth - *Methanothermobacter thermautotrophicus*; Metst - *Methanosphaera stadtmanae*; Metmp - *Methanococcus maripaludis *S2; Metva - *Methanococcus vannielii *SB; Metae - *Methanococcus aeolicus *Nankai-3; Metja - *Methanocaldococcus jannaschii*; Metvu - *Methanocaldococcus vulcanius *M7; Metin - *Methanocaldococcus infernus *ME; Metka - *Methanopyrus kandleri*; Pyrae - *Pyrobaculum aerophilum*; Pyrar - *Pyrobaculum arsenaticum *DSM 13514; Pyris - *Pyrobaculum islandicum *DSM 4184; Thene - *Thermoproteus neutrophilus *V24Sta; Pyrca - *Pyrobaculum calidifontis *JCM 11548; Thete - *Thermoproteus tenax*; Calma - *Caldivirga maquilingensis *IC-167; Thepe - *Thermofilum pendens *Hrk 5; Censy - *Cenarchaeum symbiosum*; Nitma - *Nitrosopumilus maritimus *SCM1; Naneq - *Nanoarchaeum equitans*; Korcr - *Candidatus Korarchaeum cryptofilum *OPF8; Calte - *Candidatus Caldiarchaeum subterraneum*.

In an attempt to shed more light on the evolutionary and functional diversity of RecJ-like proteins in Archaea, including their potential roles in the replication apparatus, and the origin of CDC45, we performed a comprehensive comparative genomic analysis of the DHH superfamily. We constructed multiple sequence alignments, ran HHpred searches [21] and analyzed gene context for all members of arCOGs that contain identifiable (although in some cases, apparently inactivated) DHH domains. To characterize the relationships between archaeal, bacterial (COG0608, COG2404, COG0618, COG1227) and eukaryotic (CDC45 and Prune) DHH proteins, we aligned representative sequence sets from each family and used alignable blocks of the DHH phosphoesterase domain for phylogenetic tree reconstruction (Figure 1A and Additional file 1).

The resulting phylogenetic tree consists of three major clades that generally reproduce the previously established relationships; the tree topology is consistent with the classification in COGs and arCOGs [20,22,23]. The Ppx1 clade includes bacterial inorganic pyrophosphatases/exopolyphosphatases (COG1227, Ppx1), members of arCOG1567 (some of these proteins are fused to multiple CBS domains) and the eukaryotic Prune family. All three families share an active DHH phosphoesterase domain and a distinct C-terminal domain denoted DHHA2, suggesting that all these proteins perform the same function [23]. These orthologous families additionally cluster with bacterial members of COG0618 and the archaeal proteins of arCOG01566 (some of which are fused to a Rossmann-like NAD-binding domain) and the diverse arCOG01565. All these protein families contain the DHH phosphoesterase domain and the distinct, C-terminal DHHA1 domain. Both the DHHA1 and DHHA2 domains are structurally similar to the C-ala domain present in alanyl-tRNA synthetase and several other aminoacyl-tRNA synthetases and are implicated in RNA or ssDNA binding [24]. None of these proteins have been experimentally characterized but the similarity with Ppx1 and several fusions with NAD-binding domains suggest that they are enzymes involved in as yet uncharacterized metabolic pathways.

The second clade consists of several arCOGs related to bacterial members of COG2404 (COG2404 clade in Figure 1A). All these proteins contain a (predicted) active DHH phosphoesterase domain and the C-terminal DHHA1 domain. Given that COG2404 includes relatively few bacteria, in contrast to a much greater abundance of homologs in Archaea, it seems likely that bacteria acquired these genes from Archaea via horizontal gene transfer (HGT). The member of COG2404 from *Bacillus subtilis *recently has been experimentally characterized as a nanoRNAse that degrades small RNAs [25]. A similar activity can be predicted for the related arCOGs in this clade.

The third clade (denoted RecJ) consists of bacterial RecJ (COG0608), eukaryotic CDC45, a variety of archaeal homologs including two groups (arCOG00432 and arCOG00428) with inactivated DHH phosphoesterase domains and a distinct branch containing COG1107 and the Euryarchaea-specific arCOG00429. The proteins in the latter branch contain an active DHH domain and a DHHA1 domain, and are additionally fused to a DnaJ-like domain and two distinct OB-fold domains, suggestive of a highly specialized function. The 'main' archaeal group in this clade is arCOG00427 which consists of proteins with an intact DHH domain including the experimentally characterized GAN nuclease from *T. kodakarensis*. In addition, two groups of archaeal proteins with apparently inactive DHH domains also cluster with arCOG00427.

Within the RecJ clade, eukaryotic CDC45 clusters with the archaeal homologs to the exclusion of bacterial RecJ family, at least when only the DHH domain alignment is used for tree construction and with a relatively low bootstrap support value of 59 (Additional File 1). We further attempted to decipher the domain architecture of CDC45. The secondary structure prediction obtained predicted for the multiple alignment of CDC45 using Jpred [26] is compatible with the presence at its C-terminus of a DHHA1 domain that is connected with the N-terminal DHH domain via an alpha-helical linker. Specifically, the core elements of the DHHA1 domain, which include five beta-strands and three alpha helices, were predicted in CDC45 (Additional file 2). Furthermore, an HHpred search [21] using Hbut_0430 from *Hyperthermus butylicus *(arCOG00432) as a query, in addition to the DHH domain, identifies significant similarity with the CDC45 profile for the central linker region and a fragment of the predicted DHHA1 domain, thus covering all three distinct parts of these proteins (Additional file 3). Thus, CDC45 appears to be a bona fide ortholog of the active archaeal RecJ-like nucleases with a conserved domain architecture. By implication, the roles played by these orthologous proteins in DNA replication of Archaea and eukaryotes are likely to be mechanistically similar.

The *T. kodakarensis *gene encoding the GAN nuclease belongs to a predicted operon with two ribosomal proteins (universal S15 and archaeo-eukaryotic S3) and the Pcc1 subunit of the KEOPS complex which is involved in an essential tRNA modification and possibly other functions related to translation [27]. This genomic context of arCOG00427 proteins is fully or partially conserved in many archaeal genomes (Figure 1B). *Halobacteriales *and *Desulfurococcales *lack representatives of arCOG00427 but notably possess, respectively, genes of arCOG00428 and arCOG00432 (Figure 1C), which encode proteins with apparently inactivated DHH domains and are located in the same genomic neighborhood. This conservation of the genomic context suggests that these apparently inactivated RecJ-like proteins are orthologous to the (predicted) active nucleases of arCOG00427 and might perform similar functions.

Due to the deterioration of the DHH domain in arCOG05902, the *Sulfolobales*-specific RecJdbd protein found to be associated with the GINS complex in *S. solfataricus *[12], we could not include this family into the tree. Nevertheless, the overall sequence similarity suggests that the RecJdbd proteins are derivatives of arCOG00427 (Additional file 3). Similarly, the *Methanococcaceae*-specific arCOG00433 is apparently a product of a lineage-specific duplication of a RecJ-like protein gene from arCOG00427, followed by loss of the DHH domain (Additional file 3).

Taken together, the phyletic patterns of all arCOGs related to archaeal RecJ orthologs from arCOG00427 cover all archaeal lineages except for the majority of *Thermoproteales *(Figure 1C and Additional file 4). Taking into account the genomic context in which RecJ orthologs are typically encoded in other Archaea (Figure 1B), we examined the neighbors of the genes encoding ribosomal proteins S15 and S3 and Pcc1 in all available genomes of *Thermoproteales*. In most of these genomes, two or all three genes from this neighborhood are colocalized with a gene encoding a "hypothetical protein" which belongs to arCOG05692 (Figure 1B). The arCOG05692 proteins show no detectable similarity with RecJ (or any other protein family) in standard sequence similarity searches (PSI-BLAST against nr database and HHpred search). However, using PSI-BLAST search against the archaeal subset of the non-redundant NCBI database, it is possible to detect statistically significant similarity between the RecJdbd (arCOG05902) sequences and the sequences of arCOG05692 (Additional file 3). Moreover, secondary structure prediction for these proteins is compatible with the presence of an inactivated catalytic DHH subdomain, alpha-helical subdomain of DHH domain, potential linker region and most of the core elements of the DHHA1 domain (Additional file 5). Taken together, these findings strongly suggest that, the extreme sequence divergence notwithstanding, arCOG05692 proteins are *bona fide *RecJ orthologs with an inactivated DHH domain. Thus, the only archaeal genome in which we were so far unable to identify RecJ orthologs is *Caldivirga maquilingensis *(Figure 1C and Additional file 4). The typical RecJ neighborhood is not conserved in this genome; it appears likely that *Caldivirga maquilingensis *encodes a RecJ homolog with an inactivated DHH domain that has diverged beyond recognition, at least at the sequence level. Thus, we hypothesize that a RecJ homolog is an essential structural component of the replication machinery in all Archaea.

### Evolution of the RecJ family in Archaea and eukaryotes

The findings described above allow us to propose a scenario for the evolution of RecJ homologs in Archaea and the origin of CDC45. Most likely, the last common ancestor of all extant Archaea possessed a single RecJ ortholog that was encoded in the conserved neighborhood including also the S15, S3 and Pcc1 genes. This ancestral protein was an active DHH nuclease and an essential component of replication machinery. This ancestral protein also might have performed additional functions, e.g. in repair, that required the nuclease activity. In Crenarchaeota, the DHH domain has partially deteriorated, losing the nuclease activity, and the RecJ-homolog apparently became a dedicated replication system component; the subsequent routes of evolution were notably different between the three major crenarchaeal branches (*Sulfolobales, Desulforococcales *and *Thermoproteales*), resulting in extreme sequence divergence. The archaeal ancestor of eukaryotes, the exact nature of which remains elusive, also retained the RecJ ortholog (CDC45) in which some but not all catalytic residues of the DHH domain are conserved; so far, to our knowledge, there is no experimental data demonstrating a nuclease activity in CDC45.

In euryarchaea, the RecJ gene seems to have undergone triplication. One clade evolved very fast and developed some specialized function (arCOG00429, COG1107). Two other clades (arCOG00427_I and arCOG00427_II) retained significant levels of sequences similarity. Most of these proteins contain an active DHH nuclease domain, suggesting that they remain active nucleases. Only one of these paralogs (arCOG00427_II) is often located in the conserved neighborhood with S15, S3 and Pcc1. Thermococci have apparently lost one paralog (arCOG00427_I). Methanococcales encode an additional paralog (arCOG00433) that has lost the DHH domain; the function of this protein remains obscure. These genes are located elsewhere in the genomes and it is unclear if they have any role in replication. In Halobacteria, the RecJ orthologs arCOG00428, which are encoded in the same conserved neighborhood, contain an inactivated DHH domain. Thus, inactivation of the RecJ-like nuclease that apparently became a dedicated replication protein occurred at least twice independently in different archaeal lineages. However, two alternative evolutionary scenarios are conceivable. The first one includes a duplication in the stem of Euryarchaeota that yielded a RecJ proper homolog (ancestor of arCOG00427) and arCOG00429. This duplication would be followed by another duplication of arCOG00427 at the base of the Thermococci branch. In addition, HGT of a gene of arCOG00427_I from Euryarchaeota to Korachaeaon has to be assumed. The second scenario involves duplication of the RecJ proper homolog (ancestor of arCOG00427) at the base of archaea with several independent losses of arCOG00427_I followed by a second duplication in Euryarchaeota yielding arCOG00429.

## Conclusions

The accumulating experimental data and the analysis presented here suggest that the presence of at least one RecJ ortholog is essential for all archaea and eukaryotes because this protein is most likely an indispensable component of the replication machinery. Although the nuclease activity of the DHH domain is not essential, the DNA-binding DHHA1 domain probably plays a crucial role in replication. As recently proposed for the Sulofolobus RecJdbd [28], this domain might direct ssDNA generated by the MCM helicase to the catalytic site of the primase, providing the essential coupling of DNA unwinding and replication initiation.

The observations presented here raise many questions for further experimental study, in particular:

-what is the function (if any) of the nuclease activity of the arCOG00427 proteins in replication?

-are active RecJ-like proteins involved in processes other than replication, such as DNA repair or even translation, a possibility that is suggested by their conserved genomic context?

-is there functional diversification between arCOG00427_I and arCOG00427_II and can these proteins substitute for one another or are they present together in the replication complex?

-what are the functions of uncharacterized RecJ paralogs in Euryarchaeota?

## Methods

The NCBI Refseq database [29] was used for retrieval of information on genomic context. The recent update of the arCOG database [20] that includes 70 complete archaeal genomes (ftp://ftp.ncbi.nih.gov/pub/wolf/COGs/arCOG/) was used for the analysis of phyletic patterns of the relevant genes. Protein sequence database searches were performed using PSI-BLAST [30] as described in Additional file 3. The HHpred server that performs comparison of protein family profiles using the Hidden Markov Model technique was employed for detection of remote sequence similarity [21]. Multiple alignments of protein sequences were constructed by using the Muscle program [31], followed by a minimal manual correction on the basis of local alignments obtained using PSI-BLAST [30] and HHpred [21]. Protein secondary structure was predicted using the JPRED program [26]. Maximum likelihood (ML) phylogenetic trees were constructed by using the MOLPHY program [32] with the JTT substitution matrix to perform local rearrangement of an original Fitch tree [33]. The MOLPHY program was also used to compute RELL bootstrap values.

## Competing interests

The authors declare that they have no competing interests.

## Authors' contributions

ZK, KSM and EVK designed the study; KSM performed data analysis; ZK, KSM and EVK wrote the manuscript. All authors read and approved the final manuscript.

## Reviewers' comments

### Reviewer's report 1

#### Prof. Patrick Forterre Institut Pasteur

Several essential proteins involved in eukaryotic DNA replication, such as the initiator protein Cdc6 (and ORC subunits), helicase MCM subunits, the GINS complex, or DNA primases, have closely related homologues (possibly orthologs) in Archaea. From this observation, it seems logical to conclude that the DNA replication machineries in Archaea and Eukarya derived from an already well elaborated DNA replication machinery present in the DNA-based ancestor of Archaea and Eukarya. In such scenario, homologous DNA replication proteins in these two domains are true orthologs. Going one step further, it is often conclude that all DNA replication proteins performing similar function in these two domains SHOULD be orthologs, even if they only exhibit structural similarity, having extensively diverged in term of primary sequence. In this paper, Makarova, Koonin and Kelman push this reasoning to the limit by assuming that archaeal proteins that only share similar (but very divergent) domains are finally also orthologs. They conclude that these proteins (homologs of bacterial RecJ), share in fact a most recent ancestor with the eukaryotic protein CDC45, but, for unknown reason, have extensively diverged from the eukaryotic protein, and continued to do so during the diversification of the archaeal domain. Since one of these archaeal proteins, GAN, has been shown to associate with the archaeal GINS complex in Thermococcus kodakaraensis, they conclude that a putative GINS/"CDC45/GAN"/MCM complex in Archaea is orthologous to the GINS/CDC45/MCM complex in Eukarya. This is possible. However, there are other possibilities that are not discussed in this paper. To explain why several features of the DNA replication machineries are strikingly different in Archaea and Bacteria (such as the absence of type IIA DNA and Type IB topoisomerases or of DNA polymerase alpha in Archaea, or else the absence of "archaeal" DNA polymerase D in Eukarya), I suggested a few years ago that the DNA replication machineries in Archaea and Eukarya are in fact not orthologs, but were built independently in these two domains from both homologous and non homologous proteins recruited from different DNA viruses encoding their own replication machineries [34]. From that time, a type IB DNA topoisomerase has been finally found in thaumarchaea [35], but the problems raised by Topo IIA or DNA polymerase alpha remain. In my 2006 paper, I suggested an RNA-based ancestor of Archaea and Eukarya. There are also intermediary scenarios, for instance, Archaea and Eukarya could have derived from a DNA-based ancestor, but many ancestral DNA replication proteins can have been replaced by viral ones or new ones can have been introduced later on by viruses independently either in the lineages leading to Archaea or Eukarya, or during the diversification of these two domains. Unfortunately, in that paper, the authors don't recognize the important role that DNA viruses probably played in the evolution of the DNA replication apparatus. For instance, p5, when they said that "multiple MCM paralogs have been identified in archaeal species". In fact, these MCM proteins are not paralogs (they don't originated from gene duplication in cellular genomes) but they have been introduced in archaeal genomes by viral integration [36].

Authors' response: The work of Krupovic et al. [36] and that of Chia et al. [37] clearly demonstrate that MCM genes have been independently duplicated in several archaeal lineages. Many of the MCM genes are indeed associated with mobile elements but in the phylogenetic trees published in the above papers they cluster with the 'main' MCMs from the respective archaeal groups. Thus, there is no evidence that these genes are of viral origin, they are clearly archaeal. The association of MCMs with mobile elements might lead to acceleration of their evolutionary rates and subfunctionalization, namely dedicated involvement in the replication of these elements. The evolutionary scenario leading to the MCM association with mobile elements is of major interest but currently remains unclear.

I suspect that many other archaeal DNA replication proteins entered into cellular genomes that way, and this might be the case for the proteins discussed in that paper.

Authors' response: We do not see any evidence of this. No RecJ homologs have been detected in any viral genomes, and neither have we observed any associations of genes for RecJ-like proteins with viruses or mobile elements.

The authors have used very powerful analytic tools to detect remote similarities. However, when you perform a BLAST search with ribosomal proteins, RNA polymerase subunits or DNA replication proteins such as MCM, you don't have to use such sophisticated searches. These proteins exhibit extensive sequence similarities between these two domains. The situation is strikingly different with CDC45 and GAN. Similar BLAST searches with archaeal GAN proteins fail to retrieve significant similarities with eukaryotic Cdc45 proteins. In contrast, you recover indeed more similarities with bacterial RecJ. This is in striking contrast with the situation observed with all other proteins that are truly orthologs between Archaea and Bacteria. IN ALL CASES, the archaeal protein is much more similar to its eukaryotic homologues than to its bacterial homologues.

Authors' response: BLAST generally is not a reliable indicator of phylogenetic relationships especially for diverged proteins. Clearly, evolution of the RecJ family involved multiple accelerations of evolution. To characterize the evolutionary relationships between proteins and protein families, phylogenetic analysis and not direct sequences comparison is the approach of choice.

The phylogenetic analysis performed by the authors is more a clustering than a phylogenetic analysis since the various groups analyzed are all very divergent (including archaeal "RecJ" from bacterial RecJ). For me, it is difficult to understand why these proteins should have diverged much more than other DNA replication proteins, including MCM, Cdc6 or Topo IB between Archaea and Bacteria if they are true orthologs. It is also difficult to understand why they have instead conserved some similarities with their more remote bacterial ancestor! This does not make real sense.

Authors' response: As indicated repeatedly, RecJ-like proteins are a complex family with convoluted evolutionary history, and there are pitfalls in phylogenetic analysis of such families. Nevertheless, as pointed out in the text, the tree presented here is consistent with all previously established relationships between DHH protein families. Moreover, the diverged arCOGs from Desulfurococcales and Halobactreia cluster with arCOG00427_II which is compatible with the localization of all these genes in the conserved neighborhood with S15, S3 and Pcc1. Thus, despite the divergence of these sequences, it appears likely that the tree in general accurately reflects the relationships between these families. Furthermore, grouping of CDC45 with archaeal RecJ homologs is also consistent with the presence of GINS proteins which interact with CDC45 in eukaryotes and with RecJ homologs in archaea (but not in bacteria that do not encode any GINS proteins as far as we are aware)

In my opinion, it is more reasonable to think that many variants of a large superfamily, including Cdc45, RecJ, Gan and others, emerged and diverged in the virosphere very early on, i.e. before the divergence of the three domains, and were recruited later on independently to improve the efficiency of replication forks (or for various steps in DNA repair) in various domains and lineages.

Authors' response: See the response about viruses above. We are well aware of the importance of the virosphere in cellular evolution as a whole. However, in the case of the RecJ-like protein family, the absence of any link to viruses or mobile elements, location of even the most diverged genes in the same, conserved gene neighborhoods and interaction with GINS proteins (that are as well highly diverged and so far not found in any viral genomes), the virosphere does not seem to be directly involved here.

With this interpretation in mind, I would suggest to be more cautious before concluding that, in all cases, the proteins analyzed in this study are members of a GINS/MCM/"GAN" complex functionally analogous to the eukaryotic CDC45, MCM, GINS complex. This is possible, but to be sure will require much of experimental work.

Author's response: Clearly, the conservation of the complex in all Archaea is a prediction that stems from comparative genomic analysis. However, as far as such predictions go, we believe it is a very strong one.

### Reviewer's report 2

#### Dr. Stephen John Aves (nominated by Dr. Purificacion Lopez-Garcia), University of Exeter

The authors follow up a recent report of homology between the essential eukaryotic DNA replication protein Cdc45 and archaeal RecJ nuclease homologs by performing a comprehensive bioinformatic analysis of RecJ homologs in Archaea. Sequence similarity searches, protein domain analysis and genomic context enable them to identify at least one RecJ homolog in virtually all sequenced archaeal genomes, which is a very important finding. Despite this conservation, some of these archaeal homologs contain an intact DHH nuclease domain like RecJ, whereas others, like Cdc45, have an inactive DHH domain. The homologs are also demonstrated to share other sequence and domain similarities and the authors perform a comprehensive phylogenetic analysis of the entire DHH superfamily to which the RecJ nucleases and Cdc45 belong. All these bioinformatic analyses enable them to confirm and extend the hypothesis that Cdc45 is an ortholog of archaeal RecJ proteins, and to propose that an essential DNA replication role for the Cdc45/RecJ-MCM-GINS (CMG) complex is conserved between Archaea and Eukarya.

This is a very good and comprehensive phylogenomic analysis and the conclusions and inferences are generally sound and provide a number of predictions, particularly about archaeal RecJ homologs, which will lead to useful lines of experimental investigation for DNA replication research.

I question the title which I think concludes one step too far: yes the CMG complex is conserved in all Archaea and Eukarya, but I think the evidence so far only allows the conclusion that this is likely to be a component of the DNA replication system.

Authors' response: We believe the prediction is very strong and after careful consideration have opted to keep the title as it was. This comment may serve as a word of caution..

I also question the introduction of the abbreviation RMG (for RecJ homolog, MCM and GINS) rather than expanding the existing CMG to include Cdc45 homolog, MCM and GINS. This would prevent an additional abbreviation for what is, after all, concluded to be a conserved complex in Archaea/Eukarya.

Authors' response: Yes, we agree with this point. In the revised manuscript, we speak of 'archaeal CMG complex'.

Other specific suggestions:

The Introduction should also cite Pacek et al. (2006) Mol. Cell 21, 581-7, who first provided evidence that the CMG complex is the active replicative helicase unit (the "unwindosome") in vivo. Sequence and structural homology between Cdc45 and DHH proteins beyond the DHH domain have also been very recently reported by Krastanova et al. (J. Biol. Chem. http://www.jbc.org/cgi/doi/10.1074/jbc.M111.285395) and it would be good to note this.

Authors' response: These papers are cited in the revised text (references 3 and 15, respectively).

The statement "Within the RecJ clade, eukaryotic Cdc45 clusters with the archaeal homologs to the exclusion of bacterial RecJ family" is only weakly supported by bootstrap value, and the text should make this clear.

Authors' response: Yes, this is pointed out in the revised text.

In addition there are also some minor errors and typos to correct.

Authors' response: We corrected several typos and minor errors.

### Reviewer's report 3

#### Prof. Martijn Huynen, Radboud University

I have very little comments on this article, which thoroughly examines the phylogenetic distribution and genomic context of the RecJ clade of the DHH family among the Archaea. The results presented support the conclusions.

Nevertheless:

The title is a bit of a stretch, after all the authors I) only show (new) results with respect to the DHH family, and II) no results that arCOOG00427 are a component of the DNA replication system.

Authors' response: See response to Reviewer 2.

In a sense, the article, and specifically Figure 1c, questions the validity of the arCOG database, or at least shows that it is, at least for the protein family studied here, too narrowly in its definition of orthologous groups. We have once defined a strategy to merge orthologous groups based on profile-profile hits and complementarity of phylogenetic distribution (Dutilh et al, Signature Genes as a Phylogenomic Tool, Mol. Biol. Evol. 2008) and similar implementations of this idea may have been published. It would be nice to refer to those.

Authors' response: Reconstruction of orthologous sets of genes is a difficult task, especially for gene families with complex histories of duplication and loss, and bad cases can be found in any existing systems of orthologous genes. From our extensive preceding experience, we were well aware of the complexity of this task when developing arCOGs. In the original arCOG paper, we described the strategy of merging orthologous groups based on profile-profile hits and complementarity of phylogenetic distribution (Makarova KS, Sorokin AV, Novichkov PS, Wolf YI, Koonin EV. Clusters of orthologous genes for 41 archaeal genomes and implications for evolutionary genomics of archaea. Biol Direct. 2007 Nov 27;2:3). This approach is indeed similar to the approach used by Dutilh and coworkers. However, even this, sensitive sequence comparison approach does not guarantee the proper agglomeration of all sub-COGs for highly diverged gene families. Some of these problems can be and indeed have been resolved with manual intervention, and more can be done with the now increased number of genomes. The same is applicable for splitting some arCOG on basis of the phylogenetic evidence. It is worth noting that we keep improving the arCOGs continuously, in particular, and several changes in arCOGs including the DHH superfamily proteins will follow this publication.

I do not see how the results reported in Figure 1c support the triplication of the recJ family in the Euryarchaea. Please show some more detail here, also regarding how the family was split in arCOOG00427_I and arCOOG00427_II and why the Thermococci lost a paralog instead of the duplication (triplication?) happened after the branching off of the Thermococcali.

Authors' response: Indeed, currently the existing data does not allow us to differentiate between three possibilities which we consider as more or less equally plausible:

1) Triplication in Euryarchaea with the loss of arCOG00427_I in the Thermococci and HGT of this gene to Korachaeaon from a Euryarchaeon;

2) Duplication leading to RecJ proper homolog (ancestor of arCOG00427) and arCOG00429 followed by further duplication of arCOG00427 after the branching off of the Thermococci. Again, HGT to Korachaeaon has to be assumed.

3) Duplication of RecJ proper homolog (ancestor of arCOG00427) at the base of archaea with several independent losses of arCOG00427_I followed by a duplication in euryarchaea leading to arCOG00429.

All these scenarios are mentioned in the revised text.

A detailed tree is presented in Additional File 1 along with an additional phylogenetic tree for arCOG00427.

Editorial: phosphoestarase, not "phosphoesterase"

Authors' response: Corrected.

## Supplementary Material

Additional file 1 **Aligned blocks of DHH domain used for the phylogenetic tree reconstruction and resulting maximum likelihood tree with bootstrap probability values. The separate phylogenetic tree for arCOG00427**. The multiple alignment and maximum likelihood trees include details on the sequences used for the tree reconstruction and the original RELL bootstrap values. Click here for file

Additional file 2 **The multiple alignment and secondary structure prediction for CDC45 family**. The provided data presents multiple alignment, secondary structure prediction and map of subdomains for CDC45 family. Click here for file

Additional file 3 **Results of sequence similarity searches for highly diverged archaeal RecJ homologs**. The provided table reports the parameters used for sequence similarity searches and statistical support values. Click here for file

Additional file 4 **The phylogenetic patters for DHH superfamily arCOGs**. The table shows patterns of presence and absence as well as the number of paralogs for each arCOG from the DHH supefamily. Click here for file

Additional file 5 **The multiple alignment and secondary structure prediction for arCOG05692 family**. Multiple alignment, secondary structure prediction and map of subdomains for predicted RecJ homologs of arCOG05692. Click here for file
