# Supplementary material for: The CMG (CDC45/RecJ, MCM, GINS) complex is a conserved component of the DNA replication system in all archaea and eukaryotes
Source: Biol Direct. 2012 Feb 13;7:7. doi: 10.1186/1745-6150-7-7 (PMC3307487; doi:10.1186/1745-6150-7-7)
Supplement: Additional file 1 — Aligned blocks of DHH domain used for the phylogenetic tree reconstruction and resulting maximum likelihood tree with bootstrap probability values. The separate phylogenetic tree for arCOG00427. The multiple alignment and maximum likelihood trees include details on the sequences used for the tree reconstruction and the original RELL bootstrap values. [file 1745-6150-7-7-S1.DOCX]

Aligned blocks (229 sequences in total, 83 aligned positions) corresponding to DHH domain used for the reconstruction of phylogenetic tree (Figure 1A). The MUSCLE program[[2](#_ENREF_2)] was used for construction of the multiple sequence alignment. The sequences are denoted by their GI numbers, abbreviated taxomony for major lineages, complete species name and arCOG or COG number to which this sequence has been assigned in arCOG or COG database. Eukaryotic families Prune and CDC45 are designated correspondingly.

57237711_delta_Campylobacter_jejuni_RM1221__COG2404 KIYHLSH--TDLDGYACQFIVNFYFKAIILITDLNLNLNKIFLLDHHQSSKRCATKIVYDFFSKVLELSKFVDVVNAVDIWLS

15611450_delta_Helicobacter_pylori_J99__COG2404 QVYHLSH--IDLDGYACQLVSKQFFKFLILVSDLNLNLNQIQLLDHHISTNRCATKIVYEFLKKTLWLEPLVEMVNSVDIWDT

184156154_Bacil_Lactobacillus_fermentum_IFO_3956__COG2404 RIKIFSH--NDLDGFGAPYLLQAVQETDVYIMDMTPDSEHWLVFDHHESVNPSAASLAWDWLTTRRDLAYLIELIRAYDTWDW

16078881_Bacil_Bacillus_subtilis_str_168__COG2404 MYHLYSH--NDLDGVGCGIVAKLAFGDALFITDLAVNEEKVKLIDHHKTRLTSATSLLYGYLIETNALDQFTELVRQYDTWEW

73667669_Metha_Methanosarcina_barkeri_str_Fusaro__arCOG00430 MILIYHH--DDNDGYCAAAIAGASYDEKVWLLDFTSDNMKLIWVDHHKTVEKAACILTWEFTHPILPPAAVAYIGD-KDLWKF

20092965_Metha_Methanosarcina_acetivorans_C2A__arCOG00430 TILIYYH--DDNDGYCAAAVAGNCYDEKVWLLDFTSDKMKLTWIDHHKTIEKAACVLTWEYAHPLSPPAAVAYIGD-KDMWRF

156937350_Therm_Ignicoccus_hospitalis_KIN4I__arCOG000426 NVKVLTH--WDADGIVSAAKALRKLGGKLYVMDYSMPYEGLVVLDHHAAYVPSASLVVSDYFGLYDWRDAVAIAGDLHDPWGN

124027601_Therm_Hyperthermus_butylicus_DSM_5456__arCOG000426 EPLLVHH--WDADGIASAALLERIMGAGLVVVDYGVPQHSIVYIDHHRAEYPSTSLLLAWLLG-GREHLDLALVGLVSDLPGF

11497907_Archa_Archaeoglobus_fulgidus_DSM_4304__arCOG000426 MNLIIHH--WDTDGITSAALLIKALEERVYILDLNLPYDETVFIDHHIQESPSASFVVSEYFD-WNTWTALGAVGDVGEKAFK

261402190_Metha_Methanocaldococcus_vulcanius_M7__arCOG000426 -MLIIHH--WDTDGIVSAVLAIKALNEKIYVLDLNLPQEETIFIDHHIQDFPSASFVVSEYFS-WNYLSALGAVGDIGERAFS

14521374_Therm_Pyrococcus_abyssi_GE5__arCOG000426 MRLIIHH--WDTDGITSAALLMKKLGEEIYVVDFNVPREPTVFIDHHSQYYPSASFVVSEYLNYWNEWSALGVVGDIVKAFEV

212224857_Therm_Thermococcus_onnurineus_NA1__arCOG000426 MHLIVHH--WDTDGITSAALLVRALCEKLYVMDFNVPGEETLFIDHHTQYYPSASLVVSEYFG-WNAWSALGVVGDIGERAFE

218883665_Therm_Desulfurococcus_kamchatkensis_1221n__arCOG000423 RKAVVTH--WDIDGLASNTIISRYTRSEIVVADLNPQLTIVKWLDHHLWPFTVTADISANFVNARDRFVTMLIELAFDDDWFQ

126466299_Therm_Staphylothermus_marinus_F1__arCOG000423 KRIVISH--WDLDGIAGATIANLYMNGEIWIIDLNPQAKKIYWFDHHEWPRTIASDLVARYFNANNEYVFKLIDLAFDDDFFI

124027576_Therm_Hyperthermus_butylicus_DSM_5456__arCOG000423 RIHVVSH--VDLDGLVAAALLARWARTVVVAADLAPRSLTLLWLDHHEWRSKTTAEIACSLLSCGDGYEKMIVEIARADDG--

169237034_Halob_Halobacterium_salinarum_R1__arCOG000423 DVIVVAD--PDADGLACAAILREVYGCRLFVCDVCPDRFELRWFDHHQWEEECTADVTVRSLDAPERFRELAEVTRDHDLWLK

15791276_Halob_Halobacterium_sp_NRC1__arCOG000423 DVIVVAD--PDADGLACAAILREVYGCRLFVCDVCPDRFELRWFDHHQWEEECTADVTVRSLDAPERFRELAEVTRDHDLWLK

76801365_Halob_Natronomonas_pharaonis_DSM_2160__arCOG000423 DRVVVAD--PDADGLGCVALLRVAFDADVFICDLCPDSPAVRWFDHHQWDTVCTTDVTLEAVAYPEQYAELAAVTRDHDLWLQ

119719843_Therm_Thermofilum_pendens_Hrk_5__arCOG00431 RPLALSDA-GDVDGVVSATLFKISR-YWDFVADLPCPGKAKLRADHHVTSAPASALLAYKALSLGNATATRLVELAVTDTANI

18313771_Therm_Pyrobaculum_aerophilum_str_IM2__arCOG00431 RPIALCDV-GDVDGLASAALFKKRH-TWDFVADLPCPGKAKVRADHHVTNAPAAAVLAAKALGLNDAVAKELVEAAVTDTANI

126353667_Therm_Caldivirga__arCOG00431 RPIALCDI-GDADGLTSAALFLMKY-EWDFVADLPCPGKAVIRADHHRTEAPCSAYMAAKGLNLDLKAQDLVKVAIETDTANI

15644343_Therm_Thermotoga_maritima_MSB8__COG0618 RILVVGHIMPDGDCVSSVLSLTLGLEELLVVVDASSPDRPSVVIDHHSTSFAATAQMIFRINKADSNLATLNYLGIATDTGFF

42526987_Spiro_Treponema_denticola_ATCC_35405__COG0618 NFIIAGHKEPDGDCIGSCLAMSFFLKTGLIILDCSNFDRNYIIIDHHATEAPSTTYLIQSIIEETKEEADALFFGLCTDTGFF

15606737_Aquif_Aquifex_aeolicus_VF5__COG0618 SILILTHENPDGDSLGSGLALYKFLK-VGIVVDASGFYRKRIRIDHHVGTAPATAALVYEIIKNDKDIATCIYTGLATDTGFF

15609974_Actin_Mycobacterium_tuberculosis_H37Rv__COG0618 RVGVVCHVHPDADTIGAGLALALVLDDLVVTVDIPSVDRELLVIDHHASSADSTTTMVAEILDADPRVAHCIYAGLATDTGSF

15805852_Deino_Deinococcus_radiodurans_R1__COG0618 PIVVLSHENPDGDALGSVLGLSRALRALAAVLDVDNNDPPVVNVDHHGTSKPAAAMMVADVIDASEAVATPLMLGLNTDTGNF

15924694_Bacil_Staphylococcus_aureus_Mu50__COG0618 TIIIHRHVRPDPDAYGSQLGLKYYIQALVIVCDTANAPRKLIKIDHHPANASSTSEIIYDLISHNKDIASVLYLGIVGDTGRF

195927556_Bacil_3DEV_Sh1221_Staphylococcus_Haemolyticus__COG0618 TIIIHRHVRPDPDAYGSQLGLKLYLEALVIVCDTANAPRSLIKIDHHPAEASSTSEIIFDFISHDEHVARVLYLGIVGDTGRF

16079977_Bacil_Bacillus_subtilis_str_168__COG0618 TIILHRHVRPDPDAYGSQCGLTEILRALVIVCDTANQERKLMKIDHHPNSASSVSEMIYELYLENTKAAELIYAGIVGDTGRF

21226777_Metha_Methanosarcina_mazei_Go1__arCOG01565 NILYLCHRNADPDAVSSAFALSEAIGGFVVVVDTSTKAQSYCVIDHHTTNTTSTVEIVYDILKANRRMGIGMLTGIVTDTGHF

110620792_envir_uncultured_methanogenic_archaeon_RCI__arCOG01565 NILFLCHKNADVDSLGSAYALQRLFGDIVVVVDTSTLVQRCAIIDHHTPIASSTGELVYSLYHEDAEVAFVLVLAIVTDTGHF

116754129_Metha_Methanosaeta_thermophila_PT__arCOG01565 -KIYLCHKNADPDAIGSAFALSWLFGDLVVLIDTSVSSQRYGVIDHHLDEVDSTAQIVWDIIKSPREVALALLAGIVSDTGRF

91772274_Metha_Methanococcoides_burtonii_DSM_6242__arCOG01565 NILYLCHRNADPDAISSAFALSEAVGDLVVVVDTSTSAQNYCVVDHHATNATSVAEIVYDVLICMHRMALGLMTGIVTDTGHF

73667761_Metha_Methanosarcina_barkeri_str_Fusaro__arCOG01565 NILYLCHRNADPDAVSSAFALSEAIGGFVVVVDTSTKAQRYCVIDHHTTNSTSTVEIVYNILKANRRVGIGMLTGIVTDTGHF

118431456_Therm_Aeropyrum_pernix_K1__arCOG01565 KSAVITHRNADPDAVGAALVVREVLRLIAVVVDAANLSQLKVVIDHHERGAGSSSELAVKTAVERPSVATAALGGIVYDTGRF

124027462_Therm_Hyperthermus_butylicus_DSM_5456__arCOG01565 PIVVTGHKNADPDALAAAYVIRNILRALAVVVDTASPEQDFVVIDHHESSAKANAEIVYMMARHSRQELEVLLSGIVYDTRHF

343485780_Tauma_Caldiarchaeum_subterraneum_arCOG01565 RVQVLSHWGGDADSVGSAYVMTRLLTEVYLLVDVGSPHLPIISIDHHV-KYLATAEIVHDLVEYDKEMAEALFLGIYYDTVRL

156937607_Therm_Ignicoccus_hospitalis_KIN4I__arCOG01565 KAAVVVHRHADPDAVASA-AFFWRAGEWMIIVDTASSSQEYCRVDHHAQEASSTSEIVSLLAREDERMARALMLGIYVDSKGF

126465764_Therm_Staphylothermus_marinus_F1__arCOG01565 VYGILTHKNADPDAVSSMLILRNFLVDMYIIVDTSSPIQPYIVIDHHQPRVVSNCELIYLMLRD-PLEATICITGILYDSRRF

15643353_Therm_Thermotoga_maritima_MSB8__COG1227 RVYVIGHKNPDTDSVCSAIGYAHFKNNKVILVDHN--EIILEIIDHHRLPVGSTSTIVAEFFLK-REIAGILLSGIVSDTLFF

88602447_Metha_Methanospirillum_hungatei_JF-1__arCOG01567 PTYIIGHRQPDTDSIASSVAYASLLNQSVILVDHN--EYIIEIIDHHRLPVGSTSTIIAMRYRE-KAIATMLLAGILSDTLVL

154150559_Metha_Methanoregula_boonei_6A8__arCOG01567 QIYCFGHRQPDTDSIASVLGYADFKNRPVVLLDHN--EAVVEIIDHHRLPVGATSTIITMKFRE-REIAGILLCGILSDTLGL

124485998_Metha_Methanocorpusculum_labreanum_Z__arCOG01567 SIYVIGHRHPDTDSICSAIGYAAFLNRSVILVDHN--EYIIEIIDHHRLPVGSTSTIVTRRFME-KNVAGILLSGILSDTLGL

126178441_Metha_Methanoculleus_marisnigri_JR1__arCOG01567 KIIIIGHKQPDTDSICSVIGYAELRNRSVILLDHN--EYILEIIDHHRLPVGSTSTIVASKFIE-PSTAGLLLSGILSDTMVM

39998066_delta_Geobacter_sulfurreducens_PCA__COG1227 QIYVIGHRNPDTDSIASAIAYAQFKKKALILMDHN--ELILEVIDHHKLPVGSTCTVVASLYRE-ERTAALLLAGILTDTVIL

15807559_Deino_Deinococcus_radiodurans_R1__COG1227 MLAVFGHLNPDTDAISAAMVYARLLTRKVALVDHN--ESVTRVVDHHKLPVGCTGTILLKLHRE-PQDAKLMLSAILSDTLHF

34762898_Fusob_Fusobacterium_nucleatum_subsp-_vincentii_ATCC_49256__COG1227 EILVFGHRNPDTDSICSSIAMANLRKKKVIMIDHN--EFILEVVDHHKFPVGCTSTIVYGLYKE-EKIALLMLSAILSDTLLF

89896636_Clost_Desulfitobacterium_hafniense_Y51__COG1227 KIHVVGHRNPDTDSICAAIAYARLKQRQVILVDHN--EFILEVVDHHRVPVGSTCTIVAKCYRD-KAIAGIMLGAILSDTVIF

42519099_Bacil_Lactobacillus_johnsonii_NCC_533__COG1227 KELIFGHQNPDTDAIGTAIAYSYLQNKAVMLVDHN--EPVTHVVDHHRIPVGCTSTIMWQMYNE-QDIAGIMLSAIISDTLLL

16974964_Bacil_Bacillus_subtilis__COG1227 KILIFGHQNPDTDTICSAIAYADLKNKGVILVDHN--ERVLEVIDHHRIPVGCTATILNKXYKE-KEIAGLXLSAIISDSLLF

29376170_Bacil_Enterococcus_faecalis_V583__COG1227 KILVFGHQNPDTDAIGAAISFAYLQKEQVMLVDHN--EFILAVVDHHRIPVGCTSTIIYKMFKE-AQIAGMMVSAIISDTLLF

20808804_Clost_Thermoanaerobacter_tengcongensis_MB4__COG1227 TVYVCGHKNPDTDSICSAIAYAHLKRINVILVDHN--EFILEIIDHHRLPVGSTATIINRLYEE-PKIAGIMCAAILSDTLVF

28211308_Clost_Clostridium_tetani_E88__COG1227 IIYISGHRNPDTDSICSAIAYAELKNKKVILVDHN--ERLIAIIDHHRIPVGCTATIIASMYFE-KQIAGILCAAIISDTLLL

116753556_Metha_Methanosaeta_thermophila__arCOG01567 NIYVVGHKSPDTDSVTSAITYANLKNQKVILVDHN--EVILEIIDHHKIPVGATGTIIASMYEL-KEMAGLMMAAILSDTVLF

11498362_Archa_Archaeoglobus_fulgidus_DSM_4304__arCOG01567 VVYVVGHKNPDTDSVCSAIAFAYLWNKKVALVDHS--EKVVAIVDHHKIPVGCTATVIKLLFDK-KDIAGILLSSILSDTVIF

134046489_Metha_Methanococcus_maripaludis_C5__arCOG01567 MMYVVGHKNPDSDSICSAIALAYFLDAELFLVDHA--EKLIGIIDHHKIPVGSTATVISELYFR-ADIAGILLSAILSDTLLF

150400871_Metha_Methanococcus_aeolicus_Nankai__arCOG01567 MLYITGHTNPDTDSICSAIVLSYFMDGELILVDHA--EKLIGIIDHHKVPLGSTATVISELYFN-PDLAGLLLSAIISDTVLF

15668788_Metha_Methanocaldococcus_jannaschii_DSM_2661__arCOG01567 MRYVVGHKNPDTDSIASAIVLAYFLDCEIILVDHS--EKLIAIIDHHKVPVGSTATVIAELYFK-PDLAGLLLSAIISDTVLF

19115302_Fungi_Schizosaccharomyces_pombe_972h__Prune FSFVSGNESADLDSCASSIVYAYCLQRPIYLVDHN--SLIAGIIDHHKDECGSCCTLVCRYFMP-TNLAVLALGPILIDTGNL

6321995_Fungi_Saccharomyces_cerevisiae_S288c__Prune LTICVGNESADMDSIASAITYSYCQYINSYLVDNN--DTVVGIIDHHFDVSGSCSSLVFNYWYE-MNIAPLLMGAILIDTSNM

154331313_Kinet_Leishmania_braziliensis__Prune LTVVQGNEGGDMDSIVGCIYLAILFDKSVVLYDHN--KLVVGVVDHHFDCVGSACTLITELYRE-VMCPTLLTAPIILDTVNF

71651376_Kinet_Trypanosoma_cruzi__Prune FTLVLGNDGGDMDSIIGSIYLSLYLEKNVVLYDHN--KLVVGVIDHHFDPTGSACTLVAELFQE-VPCPELLLAPIVLDTVNF

24308263_Metaz_Homo_sapiens__Prune LHVVLGNEACDLDSTVSALALAFYLAKTLILVDHH--ILVAEVLDHRPILVGSCATLVTERILQ-RQTAALLHGTIILDCVNM

17136406_Metaz_Drosophila_melanogaster__Prune LHLVMGNESCDLDSAVSAVTLAFVYAQNVILVDHH----VTEILDHRPLSVGSCATLVAQRYLA-TSVAQLLHATIVLDTINF

257386196_Halob_Halomicrobium_mukohataei_DSM_12286__arCOG01565 SCLLVVHAHADLDAVGAAVGLARTLDDCLVVLDAPSSDRSLVVIDHHEPTAGATAALVADVIDAGSKARIALAAGLFDDTSGL

18313171_Therm_Pyrobaculum_aerophilum_str_IM2__arCOG01565 RVAVITHRRADADALACAKILELVIKDLYVLADVASVSQRCVKIDHHVINRPSCTEVALMLAEEPPDVAKLAVLGIYTDTGRL

171185827_Therm_Thermoproteus_neutrophilus_V24Sta__arCOG01565 RVAIVTHRRADADALACAKVLELVLADMYVLADVASLNQSFIKVDHHAVDRPSCTEIALELAEEPPDVAKLAILGIYADTGKL

119719564_Therm_Thermofilum_pendens_Hrk_5__arCOG01565 AVVVTSHVNTDPDGLACAYLMARLVERTFILCDFSNPSQTLVVVDHHFPREPASAVIAARILMDCATLATLGIAGILYDTKK-

150401073_Metha_Methanococcus_aeolicus_Nankai3__arCOG01565 NILFLCHHNADPDAIGGALGLKYLANDIVCFVDTASLNQNIILIDHHKKNSTSVCEIVANIFKKPKNIRTALLCGIVYDTKHL

45357807_Metha_Methanococcus_maripaludis_S2__arCOG01565 DIIFLCHHNADPDAVGAAIGLKQLANKTVFLVDTSSINQDVILIDHHKKNSTSTCEIVANLFREPKNIRIGLLCGILYDTKHL

15669178_Metha_Methanocaldococcus_jannaschii_DSM_2661__arCOG01565 EVLFLCHHNADPDAVGSCVALKYLASETVFIVDTASINQEVILIDHHKKDYPSTSEIIAEIFKEPKNVRIALLCGIVYDTKHL

57640580_Therm_Thermococcus_kodakarensis_KOD1__arCOG01565 SFLLLCHHNADPDSLGSAIAFARFLKDVVVIFDTSSLEQFTILIDHHVETRTSTAEIVWELFKYDGPSVKALLAGIVTDTANF

14521710_Therm_Pyrococcus_abyssi_GE5__arCOG01565 GIILLCHHNADPDSLGSAIAFSNFLLRVVFIFDTSSLEQKLIVIDHHVEKRTSTAEIVWELFKKDEDSAKVLLAAIISDTSSF

15669829_Metha_Methanocaldococcus_jannaschii_DSM_2661__arCOG01566 PLLILTHINPDPDAIASAMALKTLAECVIAVIDTSTSKQIDIIIDHHNNEVGATASILTQYLMESRNLATALFYGIQSDTDYF

91773822_Metha_Methanococcoides_burtonii_DSM_6242__arCOG01566 ALAIVVHDNPDPDAISSALALQKIAETDIAMVDCAVPGALGIVLDHHPLNVGASATILTKYLQEDKVLATALLYGIRTDTLDF

261403474_Metha_Methanocaldococcus_vulcanius_M7__arCOG01566 PLLILTHINPDPDAIASAMALKTIAECIIAVVDTSSSKQIDIIIDHHNNEVGATASILTQYLMESRNLATALFYGIQSDTDYF

73667916_Metha_Methanosarcina_barkeri_str_Fusaro__arCOG01566 KLAIVIHDNPDPDAISSGLALKEIAKDEIALIDCSIPGVVGIVIDHHPPNFGATATIMTKYLQQSKTLATALLYGIRTDTQDF

21228311_Metha_Methanosarcina_mazei_Go1__arCOG01566 KLAIVIHDNPDPDAISSGLALKEIAKDEIALIDCSIPGVVGIVIDHHPPNFGATATIMTKYLQQSKTLATALLYGIRTDTQDF

15789552_Halob_Halobacterium_sp_NRC1__arCOG01566 SVAFVVPENPSVDALAAATGLCDLCEDGAVAV--GGGGTVVAVIRHRPADAGATATIVSRFFERDQDVATALQHGIRAATREF

222478625_Halob_Halorubrum_lacusprofundi_ATCC_49239__arCOG01566 EIAVLMHPNPDPDAMSAALGVASLAEESIVLVDHNEPRGPLAVVDHHPGDYGATASVVAEYFQQPTDTATGLLYGILTDTKHL

55378597_Halob_Haloarcula_marismortui_ATCC_43049__arCOG01566 RIAVLMHPNPDPDAMASALAVSQLVAEAVVLVDHNTARGPVAVIDHHPGDYGACATIFASYFNQPCALATGLMYGIQSDTRSL

257052273_Halob_Halorhabdus_utahensis_DSM_12940__arCOG01566 RVSVLMHPNPDPDAMAAAQGVAELAADAVVLVDHNEARGPVAVIDHHPGEYGACATIVAEYFEESPPVATGLLYGIQADTNHL

110667607_Halob_Haloquadratum_walsbyi_DSM_16790__arCOG01566 TLAIVMHDAPDPDAIASALALAELADDSIALVDHSRPGVVDIVVDHHPPNVGATSTIFADYLRRTESVATALLFGIRTDTNDF

15789844_Halob_Halobacterium_sp_NRC1__arCOG01566 RLGVFMHDNPDPDAIAAAVGLQRIAEDAVALVDHSGPGVVDIVIDHHPPDTGATSTILVEYLRGRTAVATALLYGIRVDTDDF

76801801_Halob_Natronomonas_pharaonis_DSM_2160__arCOG01566 PLAVVAHDNPDPDAIGSAIGLKRIAEGGIALVDHSRPGAIDIVIDHHPPDVGATCTLVAGYLREDENLASGLLYGIWTDTQSF

169237387_Halob_Halobacterium_salinarum_R1__arCOG01566 SLAILCHDNPDPDTLASALALEAIAKGLVAFVDHGIPGQPDIIIDHHPTAVGATATLLTRYLEIEERLATALLFALHRETLDF

16120127_Halob_Halobacterium_sp_NRC1__arCOG01566 SLAILCHDNPDPDTLASALALEAIAKGLVAFVDHGIPGQPDIIIDHHPTAVGATATLLTRYLEIEERLATALLFALHRETLDF

88601913_Metha_Methanospirillum_hungatei_JF1__arCOG01566 IFGIVTHTNPDPDAIASAMALAAIAKTHIALVDSKGPGAVDIIIDHHQENVGACATILTEYIKEDEKVATALLYGIRADTKQF

124485576_Metha_Methanocorpusculum_labreanum_Z__arCOG01566 TLGIITHKNPDPDAISSAMALSVIANNYLALVDCVGPGMINIIVDHHSADAGATASILTQYLQEDTKVATALFYGIRADTHEF

154151814_Metha_Methanoregula_boonei_6A8__arCOG01566 ILGIITHKNPDPDAIASAMALAEIARTYLAMVDCSGPGAIQIIIDHHKDGIGATASILTQYLQEDKRVATALLYGIRTDTKEF

126178347_Metha_Methanoculleus_marisnigri_JR1__arCOG01566 TLCIVTHLNPDPDSISSAMALSMIARDYIALVDSSGPGVVNIIIDHHKNGVGATASIMTQYLMENKSVATALLYGIRADTRDF

11499611_Archa_Archaeoglobus_fulgidus_DSM_4304__arCOG01566 RLGIFTHDNPDPDSMSSAYALREIAKDAFAIVDSSGPGVISIVIDHHPADVGATATILTEYIKESKILATALFFGIKSETDEF

20093566_Metha_Methanopyrus_kandleri_AV19__arCOG01566 TLAIIPHNNPDPDSIASAVALQEIASDAIAVVDTPVLPRILVAVDHHDSEVGAASTILTQYLKIDRRIATALLYGIRTDTLNF

110621295_envir_uncultured_methanogenic_archaeon_RCI__arCOG01566 RIGIVVHDNPDPDSIASALALKNLAENVLAMVDCNTPGRINIIIDHHAPDVGATSTIMTRYVQESSNLATALLYGIRTDTSEF

15790097_Halob_Halobacterium_sp_NRC1__arCOG01566 GFAIITHDSPDPDSIAAAAALQAVARDTIALVDPAKAGAVDIFIDHYELSVSATSTILTKYIQESQTVATALLYGIRAETLDF

76802490_Halob_Natronomonas_pharaonis_DSM_2160__arCOG01566 TLAVLAHRSPGPDSIASAVALQAIAEDTLALVDCAKATEVDILIDHFEPNVSSTSTILTKYIQESEEVATALLYGIRAETLDF

14520471_Therm_Pyrococcus_abyssi_GE5__arCOG01566 SLLIVMHDNPDPDSMASASALAVIAQDAIAIVDAQPNGNVEIIVDHHQIDVNATSSIMVEYLKATDTLATALFYGMYIDTKKF

57641204_Therm_Thermococcus_kodakarensis_KOD1__arCOG01566 TLGIIMHDNPDPDSIASATALAAIAQPFLALVDCQPNGNIKIVIDHHQVEVNSASSILVEYLRGTPALATALFYGIYVDTKKF

20095028_Metha_Methanopyrus_kandleri_AV19__arCOG000427 AVRLYVH--NDADGLTAGALMAFTLRGPTVVVDMGSGILPVLVIDHHEITEASASTVAYLLCRRETCLPKAALVGAYGDNQAG

88603751_Metha_Methanospirillum_hungatei_JF1__arCOG000427 SVEILCH--HDADGIAAGSIMAMALYRPLLLCDLGAGLA--MVIDHHLPTDLCAAGTAYLVANANRDLAGLTLLGVIGDGQTL

154151775_Metha_Methanoregula_boonei_6A8__arCOG000427 FVEVYAH--HDADGIAAGAILCHAMLAVSLLCDLGSGKE--IVVDHHIPRELSAAGMAYIVAQENRDLAGLVIPGIIGDGQAF

48477317_Therm_Picrophilus_torridus_DSM_9790__arCOG000427 YVRVLAH--YDGDGVSSAIILTNALKVFTIMVDAGSDQANYVILDHHFHTEACGATMAFLMALTNKDLLPFFVSGVMADKQDI

16082593_Therm_Thermoplasma_acidophilum_DSM_1728__arCOG000427 FLRVIVH--YDGDGTSSAIILTNMLKDTTIVADAGSDQLNIIVLDHHFYHEACGSTMSYMMALVNSDLFPFFMSGLIADKQDL

13542040_Therm_Thermoplasma_volcanium_GSS1__arCOG000427 YLKIITH--YDGDGTSSAIILTSMAKDLVIFADAGSDQLNIIVLDHHFYRGACGSTMAYIMALINADLFPFFMSGLIADKQDL

118193829_Cenar_Cenarchaeum_symbiosum_A__arCOG000427 --MVTTH--MDCDGIASGGIMAKALSDLHVIMDLGSGVSGWFVLDHHPVSEASAGTMAYLAACANTDLAPCALVSALGDRQDS

161529017_marin_Nitrosopumilus_maritimus_SCM1__arCOG000427 SIFVTTH--IDCDGLTSGSIITKALIDFHIVTDLGGGFGNWIVLDHHQILEICAGGMAYLASMANSDLSSIAVVSALGDRQDQ

119719428_Therm_Thermofilum_pendens_Hrk_5__arCOG000427 WVLVVSH--YDADGLSSLSLMLYFLWDYAIFLDLGSGYKNILLIDHHVPEEASASTLTYLLLRSVEKMVHVALVGALGDRLDL

170291031_Candi_Candidatus_Korarchaeum_cryptofilum_OPF8__arCOG000427 HVRVISH--IDADGLSSAGIFISILKDLYIFLDLGSGEVRVFVVDHHRPRDVSSSGLCFLLYREDYSMVPVALTGALGDLQEN

84488840_Metha_Methanosphaera_stadtmanae_DSM_3091__arCOG000427 VVRIISH--NDADGLTSAGIFANAIKKLFIFSDMGSGNLDVIIADHHQVKEVSGSGLSYLSVHGYYELAGLALTGAYGDMQFK

148643253_Metha_Methanobrevibacter_smithii_ATCC_35061__arCOG000427 VIRIISH--NDADGISAAAVLANALKDLVIFSDMGSSFVDVIIADHHQVRDLSGAGSSYLAVRDKKHLAYFALIGAFGDMQGQ

15679421_Metha_Methanothermobacter_thermautotrophicus_str_Delta_H__arCOG000427 IIRIISH--NDADGLSAAGVVARAISSLFFFCDMGSAYLDVIVADHHQPRDLSASGTAYLATRLNRKTAPLALVGALGDMQYT

150400577_Metha_Methanococcus_aeolicus_Nankai3__arCOG000427 LIRIITH--NDADGLTSGAIIIKLLTVLYIFSDMGSGQINSIILDHHPTNEISASGVCYLVAREYYDLSVLAITGAIGDMQHL

45358110_Metha_Methanococcus_maripaludis_S2__arCOG000427 LIRIVTH--HDPDGLTAGSIMLKTLLKMFIFCDMGSGQINAVILDHHPPKEISASGVCYLIARLYYDLATVAVVGAIGDMQHL

261402256_Metha_Methanocaldococcus_vulcanius_M7__arCOG000427 YIRVITH--HDTDGLSSGGILVKMLLPLFIFADMGSGQINAIILDHHPPKEITASGVCYLVAREYYDLSVLAIVGIIGDMQYS

15669018_Metha_Methanocaldococcus_jannaschii_DSM_2661__arCOG000427 YIRVITH--HDTDGLSSGGILAKMLMPLFIFADMGSGQINAIILDHHPPREITASGVCYLVAREYYDLSVLAIVGIIGDMQYN

45359245_Metha_Methanococcus_maripaludis_S2__arCOG000427 KILVCTH--IDTDGINSRIILENVLEDLIIFADLGSGQIKIIILDHHIVKEICGAGVCYLFAKIFTDLAKYAVLGAVGDVQSL

261402773_Metha_Methanocaldococcus_vulcanius_M7__arCOG000427 KVLIATH--IDTDGLTSRAILQKLSEDLTIFADLGSGQLKVIILDHHQP-EICGAGVSYLFAREWTDLAKYAVLGAVGDVQNI

15669167_Metha_Methanocaldococcus_jannaschii_DSM_2661__arCOG000427 KILIVTH--IDTDGLTSRAILQKLAEDLIIFADLGSGQLKIIILDHHQP-EICGAGVSYLFAKAWIDLAKYAVLGAVGDIQNI

15678192_Metha_Methanothermobacter_thermautotrophicus_str_Delta_H__arCOG000427 TVTVYSH--TDCDGITAATVLSKVLEDLTIFSDLGSGQRRVLILDHHPPTHVSGGGLTYLLAREYRDLSWMGLLAAVGDMQNI

84489981_Metha_Methanosphaera_stadtmanae_DSM_3091__arCOG000427 DITVFTH--TDCDGITAGSILSSIFDDLTIISDLGAGQNSVIVLDHHPPYTISGGGLSYLLAKSFYDLSWMGILSAVGDMQNS

11498306_Archa_Archaeoglobus_fulgidus_DSM_4304__arCOG000427 FIRIYTH--HDADGISAGAIIAKALLELLLFADMGSGYADVVILDHHIPFELSASGVCYFFANENRELASVALIGALGDKQRM

116753443_Metha_Methanosaeta_thermophila_PT__arCOG000427 RVVVVSH--DDADGISSAGLICNALA-PIVFCDMGSGQPEVFVLDHHKPFEISASGVVYSVVRKNRDLAGLALVGAIGDRQAM

110619811_envir_uncultured_methanogenic_archaeon_RCI__arCOG000427 FIRVVSH--HDADGITACGIICHALLVPVIFCDMGSGQPDAIILDHHVPSELSGSGVAYALARINVDLAGLAISGAIGDKQKM

91774252_Metha_Methanococcoides_burtonii_DSM_6242__arCOG000427 HARVISH--NDADGISSAAIICQALLDIVIFCDMGSGQPDTIVIDHHKPVHLCGATTTYLVAKGNVDLAGIAIAGAVGDKQLF

73669607_Metha_Methanosarcina_barkeri_str_Fusaro__arCOG000427 FVRVVSH--NDADGLTSAGIMAQALLELVIFCDMGSGQPDVIVLDHHQPTDISASGTCYLVARENVDLAGLALAGAIGDKQLF

76802243_Halob_Natronomonas_pharaonis_DSM_2160__arCOG00428 FVRLVAT--ADGDALAAAGLLASGLD-------------DGGDVTVSE-AAEIVRSLAA-----EAVDATLALAGAYAGYEP-

55378210_Halob_Haloarcula_marismortui_ATCC_43049__arCOG00428 FVRLVSD--ATGEALAATGLLARALD-------------TQLTADVTL-AFETARELGT------AD-PALALAGTIAGDV--

15789951_Halob_Halobacterium_sp_NRC1__arCOG00428 FVRVTAA--PTGGCVVAAGLLAGVCD-------------EAVAADASV-AFALAREVGT-----HPD-PVVTAAGAVDGVEP-

257051809_Halob_Halorhabdus_utahensis_DSM_12940__arCOG00428 LVHLAPV--ATGDAIAATGVLARALD-------------ASQDADLTL-AFGVARSLDAPTTD-ATDVVALAAAGVVAGDEP-

21226315_Metha_Methanosarcina_mazei_Go1__arCOG000427 FVRVISH--NDADGLTSAGIMAHALLELVIFCDMGSGHPDVVIIDHHQPTDISASGTCYLVARENIDLAGLAIAGAVGDRQLF

14520284_Therm_Pyrococcus_abyssi_GE5__arCOG000427 TIRIISH--RDADGITSAAIVAKALAKVFIFSDLGSGSLTVIILDHHPPRDLSGSGVCYFFARENKDLAYIAIVGAVGDMQEN

57641187_Therm_Thermococcus_kodakarensis_KOD1__arCOG000427 TIRLISH--RDADGITAGAILAKAVAEIYVFSDLGSGSITVVVADHHPPRDLSGSGVAYFVARENRDMAYVAIVGAVGDMQEI

88603522_Metha_Methanospirillum_hungatei_JF1__arCOG000427 EVTIISH--IDADGISSEAILAQALSSLKIFSDLGSGQQRTIIIDHHVS-KMSAAGVSYLLARENTDLAKLAIIGNIGDMMDR

124486165_Metha_Methanocorpusculum_labreanum_Z__arCOG000427 AATLISH--IDADGITSEAIISQAVSTLKIFTDLGGGQQKVLILDHHISTKCSAAGITYLVARKNKDLAKLSVVGNVGDMMAR

126179628_Metha_Methanoculleus_marisnigri_JR1__arCOG000427 SVTIVSH--IDADGISTEAILAQALTSLKLFADLGAGQQEVLILDHHVGTRMSAAGIAYLVAKAGIDLAKLAVVGNVGDMMAR

154151369_Metha_Methanoregula_boonei_6A8__arCOG000427 EITIISH--IDADGISCEAILSQAIASFKIFTDLGAGQQDVLIVDHHVSTKMSAAGVSYLIAKANIDLAKLAVIGNVGDMMAR

219853096_Metha_Methanosphaerula_palustris_E19c__arCOG000427 EVTIISH--IDADGITSEAILAEAVQSLKVFVDLGAGQQEVLIADHHVATRLSAAGVAYLIAKENTDLASVAVVGNVGDMMAR

16081637_Therm_Thermoplasma_acidophilum_DSM_1728__arCOG000427 KIIIVSH--IDADGLSSSAIAKTSLDGFKWFVDLGSGSMECLITDHHLPYEISGSGTTFLVSMANADMVVPAIVGAIGDVQDA

13541834_Therm_Thermoplasma_volcanium_GSS1__arCOG000427 KVVIVSH--NDADGLSSSAIAKVTLDGFKWFVDLGSGSMECLITDHHVPYDISGSGCTFLVSLANLDLVKPAIVGSVGDVQDA

15791135_Halob_Halobacterium_sp_NRC1__arCOG000427 SVLVASH--IDADGLTSAGIASTALEGTVLFTDFGSGQLTPVIADHHQPTELSGAGATYVLARAATDLAALAVVGAVGDRQTV

76800756_Halob_Natronomonas_pharaonis_DSM_2160__arCOG000427 SVLLASH--IDADGLTSAAVAATALEETVLFTDFGSGQLTPVIADHHQPSELSGAGAAYLLARANRDLAALAVVGAVGDMQAS

55379663_Halob_Haloarcula_marismortui_ATCC_43049__arCOG000427 RVLLASH--IDADGLTSAAIATAALSDTVLFTDFGSGQLEAIIADHHQPSELSGAGAAYVLARANRDLAALAVVGAVGDMQAV

116754737_Metha_Methanosaeta_thermophila_PT__arCOG000427 DVLVVSH--IDADGLTAAGVICTALSDLVIFTDLGSGMIRAVIADHHRPTHISGSGTAFILANANEDLSALAVVGAVGDLQDL

110621959_envir_uncultured_methanogenic_archaeon_RCI__arCOG000427 ELLVVSH--IDADGLTSAGIICTALQRTVIFTDLGSGAITAVIADHHQPTDISGSGVTFLLALKNEDLSSLAIVGAVGDLQDM

91773470_Metha_Methanococcoides_burtonii_DSM_6242__arCOG000427 SVYVVSH--IDADGLTSAGIICKALEELVIFTDLGSGMINAVIADHHQPYELSGSGMTYILANANKDLADLAIVGAVGDLQHL

73669259_Metha_Methanosarcina_barkeri_str_Fusaro__arCOG000427 SVHVVSH--IDADGLTSAGIICTALENIVIFTDLGSGMCHAVISDHHQPYELSGSGSTYLLASANQDLSSLAIVGAVGDMQHL

343484652_Tauma_Caldiarchaeum_subterraneum_arCOG00427 FIDIFTH--NDADALSSAGIIAAALRKCIIFTDMGSGYTRSVIFDHHEPREISASGVSYFVAKANIEYSPIAVVGALGDLQDK

11498342_Archa_Archaeoglobus_fulgidus_DSM_4304__arCOG000427 EVLVVTH--IDADGITAGTIAHTSLLTFVWFTDIGSGVLEFAITDHHLPFELSGATTTYLVSKYNSDLLPISIVGAVGDLQDS

118431341_Therm_Aeropyrum_pernix_K1__arCOG00432 VVRIYSY--PSRDSLVAGAYLFKRLL--SPTVLLGYDSLPVFAVSSGELGSVSAAAAIASSEVVGSWDVMVALAGCMAGRYVE

156937654_Therm_Ignicoccus_hospitalis_KIN4I__arCOG00432 FVRVYAS--PTFEELVAASQLQQTLE-QSPLITLGM---GPTLEIVPSQANSSTIVIKLLEERFDDHKIFSIIASYAAE----

126465742_Therm_Staphylothermus_marinus_F1__arCOG00432 YVRIVPR--IDLDSLVASSILFENLNKDEPTILINLPKT----------ESVSAYVSYYLDKIYIYDKILSIATGIYQSLDTV

218884648_Therm_Desulfurococcus_kamchatkensis_1221n__arCOG00432 SVRIIPD--TSLDSLIASSILLKTLRRDTPAILINLPPL----------YSVAGFIASFLDDLYWWDKVLSIIAAFYRDLYVF

124027321_Therm_Hyperthermus_butylicus_DSM_5456__arCOG00432 FVRLYVF--PSIDAALAAAIVVNVLQ-VEPSLLIGYPASPSALIGYGEQSSVAALTAAVLSEVTPFM-IYGVIAGYWRELDRG

21228072_Metha_Methanosarcina_mazei_Go1__arCOG000427 SVHVVSH--IDADGLTSAGIICTALEELVVFTDLGSGMCNAVISDHHQPYELSGSGSTYLLASANRDLSSLAIVGAIGDMQHL

88604056_Metha_Methanospirillum_hungatei_JF1__arCOG000423 KVVHLTH--NDFDAVGADAVHRIRFRDTLSISDLSYRKGRIEWRDHHRWTGRCACGICASDLTPDDVIAQEIALVVCYDLWKH

126179958_Metha_Methanoculleus_marisnigri_JR1__arCOG000423 NVVHLTH--NDLDAVGSDAIHRRTYGDLLSISDLGYQQGRIEWRDHHRWVSTCATGIVARDLAPGDPVAEEIARVVCYDLWKH

126466353_Therm_Staphylothermus_marinus_F1__arCOG000423 NYIIVTH--TDMDGVGSAALYIYLHNKNIALMDLGMNKNDIEWFDHHVWRSTCAVGVVAKYARQDEDFVSELVRGVCGDLWRF

218883475_Therm_Desulfurococcus_kamchatkensis_1221n__arCOG000423 NTLIITH--TDMDGVASAALYIYLNNSNIVITDIGVNPTRIYWFDHHVWESTCATGVVYKYSKHNEDFVSNLVNGVCGDLWRF

146304290_Therm_Metallosphaera_sedula_DSM_5348__arCOG000423 DYYAIVH--NDFDGTASASVYARAVNSSVMIADLGINESTIQWFDHHVWVTTCGAGVVNKVMNPNDEVSRRLASADCVDIWLH

15899201_Therm_Sulfolobus_solfataricus_P2__arCOG000423 EYYAIVH--NDFDGTASAAVYARAIKYNVMIADLGINASNVEWFDHHVWTSTCGAGVIYKYKNPNDEFSRRLSSADCVDIWLH

14600466_Therm_Aeropyrum_pernix_K1__arCOG000423 HLFIITH--TDLDGIGAGAVAVRLLGDLIIISDLGANREAVKWFDHHIWTSTCATGVVARYLTPGDGFIKQFVDAVCADLWKW

156938093_Therm_Ignicoccus_hospitalis_KIN4I__arCOG000423 ---MVHH--NDFDGIMGAVALFRLHSDLLYVVDIGPNDHKLVWMDHHKWRSTCGAGLAARYAKELCDCCEELVELSCIDLWIR

124027337_Therm_Hyperthermus_butylicus_DSM_5456__arCOG000423 KVHIVTH--TDLDGVASAAIYLRLSGNRIVIMDLGPNTSAVEWYDHHRWTTTCAAGVVARYAPKVDDYAEKLAAITCADLWRW

15920800_Therm_Sulfolobus_tokodaii_str_7__arCOG000423 DYYAIVH--NDFDGTASAAVYARAVNNNIMIADIGINASKIQWFDHHVWTSTCGAGVIVKYLNPNDEFSKKLTSADCVDIWLH

170289985_Candi_Candidatus_Korarchaeum_cryptofilum_OPF8__arCOG000423 KRLIITH--GDVDGILSAAIAIRRLGDELLILDIGLNKSRVYWCDHHRWPSQSNARLVLEWLGG-GDFERRLVEIADADTGKY

119719797_Therm_Thermofilum_pendens_Hrk_5__arCOG000423 IYAFVLH--GDLDGLSATATVAAALKEGIYIVDLAIDADRVTWIDHHPSEAASASTIARSFLDRPAFFEKIITIGEVAD----

218884295_Therm_Desulfurococcus_kamchatkensis_1221n__arCOG00425 RVFVAGD--WDADGVVAAALIVYSQEDLAVFLDIPYSEYQIVFIDHHITHKMPTAGLVLWELEKHQRLRSFVEVVKYMDMGK-

126466085_Therm_Staphylothermus_marinus_F1__arCOG00425 RVFVGGD--WDADGVVAAALIVYSQEDLVILLDLPYTDTKIMYIDHHLSRRMPTAGIVEKLLVEHSRLKSFVEVVKYMDSGK-

124027252_Therm_Hyperthermus_butylicus_DSM_5456__arCOG00425 PVLVIAD--WDADGATSAAMIYYAQYDVIAVLDIPLTERRLVYIDHHFSGHKPTAILTYHLLRSTPRLQAFMNAVGVLERSRR

14601903_Therm_Aeropyrum_pernix_K1__arCOG00425 -VVVVAD--WDADGVVSAAEIYYSQKDIVVILDIPYTDESILYFDHHDSGVMPTSLIVRNTLEATPRLNNFVKGVAVLEGGG-

218884316_Therm_Desulfurococcus_kamchatkensis_1221n__arCOG000424 --MILVH--GDSDGVASGALAYNFYKDNIFIADIALNEVEVVYIDHHPETCCSASELTYIFLHEDPEYSRIALYGAIGDYLDE

126466055_Therm_Staphylothermus_marinus_F1__arCOG000424 RWVILVH--GDSDGVCSGALIYRYLSDNIFIADIALSEHEIIYIDHHPETCCSASELTYRFLEEIQEYSRIALYGAIGDYLDE

119720423_Therm_Thermofilum_pendens_Hrk_5__arCOG000424 AGVILAH--GDSDGVTAAAIAKSVYRSPVIILDVAVDEAEVVYVDHHPGEGPCAAELAYRFFKPPREMSRVALYGAIGDHALG

145591097_Therm_Pyrobaculum_arsenaticum_DSM_13514__arCOG000424 RLTILAH--GDADGVCSAALVKAALRDVYIV-DVAIDEKRVVYIDHHPLEGPSASELTYRKLGGPPSYSRVALYGAISDYMDY

171186307_Therm_Thermoproteus_neutrophilus_V24Sta__arCOG000424 VLTVFAH--GDADGVCSAAVVVAALADVYIV-DVAIDEKRVVYVDHHPLVGSSASELAYRLLGGPKSYSRVALYGAISDYMDH

110621895_envir_uncultured_methanogenic_archaeon_RCI__arCOG000424 RTIIFTH--ADSDGICAGALALAAF-DQAVITDIAVNISKVIYLDHHPLTASSASEMAFRHFKESPDMSRVALYGAIGDYRDD

73667573_Metha_Methanosarcina_barkeri_str_Fusaro__arCOG000424 KTLILTH--GDSDGICSGAIAKTAY-KTLIVCDIAIDERTLYYIDHHPLIEACSSELTYRLFEDDRDMRRIAIYGAIGDFCDN

21228818_Metha_Methanosarcina_mazei_Go1__arCOG000424 KTLILTH--GDSDGICSGALAKSAF-ETLIICDIAIDERNLYYIDHHPLTEVCASELTYRVFEDSSEMKRVAIYGAIGDFSDN

118194530_Cenar_Cenarchaeum_symbiosum_A__arCOG000424 KTVCLSH-KEDTDGISSAALIRQAFGKSLYICDLGLSKKAVTYIDHHDLVNECTTVQVYEKFKSDEHAPFIAACAAVTDYMED

161528606_marin_Nitrosopumilus_maritimus_SCM1__arCOG000424 KVICISH-KEDADGISSAALIRQAFGKSLYICDLGLSKKSITYIDHHDIINECTTVQVYNAYKSNEHATFVATCAAITDYMED

16079816_Bacil_Bacillus_subtilis_subsp_subtilis_str_168__COG0608 KIMIYGD--YDADGVTSTSVMLHTLQSLIITVDTGIAAVDVIITDHHEPKELAGVGVAFKLAHAPDELLDLAAIGTIADLVPL

15924626_Bacil_Staphylococcus_aureus_Mu50__COG0608 KILVYGD--YDADGVTSTTILVTTLQTLIITVDNGIQGHDVIVTDHHEIQQLCGAGVAYKLAQAPDYFKALVAIGTIADLVSL

21283315_Bacil_Staphylococcus_aureus_MW2__COG0608 KILVYGD--YDADGVTSTTILVTTLQTLIITVDNGIQGHDVIVTDHHEIQQLCGAGVAYKLAQAPDYFKAIVAIGTIADLVSL

15965474_Alpha_Sinorhizobium_meliloti_1021__COG0608 KVVIFGD--YDVDGAASSALMARFLRELIVTVDCGSTSHDVVVIDHHQVGHLCAAGVVYLVLVNLLSLLDLVALATVCDVVPL

16130794_Gamma_Escherichia_coli_str_K12_substr_MG1655__COG0608 RIIVVGD--FDADGATSTALSVLAMRQLIVTVDNGISSHPVIVTDHHLPKSLAGVGVAFYLMLALAELLDLVALGTVADVVPL

33865740_Chroo_Synechococcus_sp_WH_8102__COG0608 ALAVCGD--YDADGMTSTALLLRALIRLLVTVDNGVAAVDVIVTDHHSIRGLAGVGLAYVLASSAERLGRMDAIQVARDLFCI

32475516_Planc_Rhodopirellula_baltica_SH_1__COG0608 PIVIYGD--YDADGMTGSAILVNCLRQMIISVDCGIASLTLIITDHHQIGELCGAGVAFKLAWSLMQSLSLAAIGTIADVVPL

19703716_Fusob_Fusobacterium_nucleatum_ATCC_25586__COG0608 KIYIYGD--YDVDGITSVSLLYLAFSDLVISVDCGINSIDFIITDHHEIKYLAGVGTAFMLIYALEKYLDIVAIGTVADIVPL

15607096_Aquif_Aquifex_aeolicus_VF5__COG0608 RIIIYGD--YDVDGITGTAILYRVLKDFLITVDNGTSAVETVVIDHHNVKALSSSALSFYLGSAPRNFLDLVALGLLADYMPV

21465637_Deino_Thermus_thermophilus__COG0608 RIRVHGD--YDADGLTGTAILVRGLADLFLTVDCGITNHEVIVTDHHTPEKPTGAGVAFLLLWAPLEYADLAAVGTIADVAPL

126179497_Metha_Methanoculleus_marisnigri_JR1__arCOG000429 PIILRHH--ADADGICAAVAVEQAVTPMILLMDNGSTEEPVMVVDHHHPDYGITAGMLGTEVARENQIRHLPAIAGLGDRSEA

124485558_Metha_Methanocorpusculum_labreanum_Z__arCOG000429 PIIIRHH--ADADGICAAVSVETAVTPLILLMDNGSTEEDVIVADHHHPDFGVTAGMLGTEIARESRILHFPAVAGVADRSEA

20093597_Metha_Methanopyrus_kandleri_AV19__arCOG000429 PVLIRHH--ADADGISGGVALEEAILPLLVLLDIGCTEEDVLVIDHHYPGKNIPAGVLAVEIAREDRIKHLPAVACLGDHAES

14521821_Therm_Pyrococcus_abyssi_GE5__arCOG000429 PIIIRHH--ADTDGYTAGVALETAIVPLIVIVDNGGTSEKIVVIDHHDPYYELTAGMLATEVAREDKIKHLPAIAGTGDRSKA

57640090_Therm_Thermococcus_kodakarensis_KOD1__arCOG000429 PILLRHH--ADADGYTSGLALEYAIVPLVVIVDNGGTSEPIVVIDHHDPYYELTAGMLATEVAREDKIKHLPAIAGTGDKSKA

15669384_Metha_Methanocaldococcus_jannaschii_DSM_2661__arCOG000429 PIIIRHH--ADTDGYCGGIALEKAILPLIVLIDNGSTDEEVIVIDHHFPDSNLTAGVLGTEIAREDEIKHIPGIAVVGDHAKG

150400655_Metha_Methanococcus_aeolicus_Nankai3__arCOG000429 PIILRHH--ADTDGYCAGLALEKAIIPLVILTDNGSTHEDVVVIDHHYPDSNLTAGALATEVARTDDIKHLPGIAMVGDHAKG

150399375_Metha_Methanococcus_vannielii_SB__arCOG000429 PVIVRHH--ADTDGYCGGIALEKAIIPLIVLVDNGSTDEEVVVVDHHFPNSNLTAGVLGTEVARTDLISHLPGIAVVGDHAKG

45358877_Metha_Methanococcus_maripaludis_S2__arCOG000429 PVIIRHH--ADTDGYCGGIALEKAIVPLIVLVDNGSTDEEVVVVDHHFPDSNLTAGALATEVARTDLVEHLPGIAVVGDHAKG

15678788_Metha_Methanothermobacter_thermautotrophicus_str_Delta_H__arCOG000429 SILVRHH--ADADGICAGVAIEKAVLPLLVLLDNGSTEEEIVVVDHHYPDSQITAGSLSVEIAKAERILHLPGIAAVGDHANS

76802079_Halob_Natronomonas_pharaonis_DSM_2160__arCOG000429 PVVVRHD--ATTDGYVAGAAIERAVLPLFVFAAAGSTAAPRVVLDGRPV--GRTSATVAAAVAARADLEHLPAVSYWDETPTV

159476804_Chlor_Chlamydomonas_reinhardtii__CDC45 VMCFAAC--TDADSVCASQQLMNLFNKTVVLINCGATDDRIVIIDHHRPGYGKPSSLLLFALVNDNFHLWCAIVGLTQLLFHQ

302829318_Chlor_Volvox_carteri_f_nagariensis__CDC45 VLCLAAS--VDADSVCASQLLLNLFNKTVLLLNCGATEDRIVVLDHHRPGYGKPSSLLLFSLCHDNFHVWCAIVALTQLLFQQ

167523405_Choan_Monosiga_brevicollis_MX1__CDC45 VLIVAA---MDVDAACACGILTRLFECAAILLNVGASYDTIYMFEAHRPYHTTPISVLLWHLVMSNDSLWRAMVALVAFVEDR

145526286_Cilio_Paramecium_tetraurelia_strain_d42__CDC45 VCIIVS---LDVDAICALRILTSLLNKSIILLNCGNVYDKFFIFDSHKPYYSRPSSTVVYTLAQCNDHLWYAILGLTAYIHMR

146185831_Cilio_Tetrahymena_thermophila__CDC45 VFIFVA---FDADSLCSLKILTGLLKQSMIFINCGGVLDKAYIFDNHRPYYGKATSMLMYKICQNNNYLWYTILGATLLIHGK

145345736_Chlor_Ostreococcus_lucimarinus_CCE9901__CDC45 VLVYVST--ADVDALCAFRTLKLMLRRAIVLINCGGTEDRAYVFDSHRPFYGRSSGMVMYDIAYNYLPLWLAVLSMTQYLHQR

168017943_Strep_Physcomitrella_patens_subsp_patens__CDC45 LLIFPSA--SDVDSLCALKIVTNLLVIVVFLINWGASRNQVFVVDSHRPFHGRPSGCLMFDIAHTNELLWLAGVALTQFVHER

302815970_Strep_Selaginella_moellendorffii__CDC45 LHILPST--VDADSVCALKIFRSILNVVVLLINWGGTQDLVFVVDSHRPFHGRAAGRQVYDISHTHELLWLACVSLTQFVHER

15230255_Strep_Arabidopsis_thaliana__CDC45 LLIFPST--SDVDSLCALKVITHILEVTILLINWGCHRDRVFVVDSHRPFHGKPSGCLLFELSHTNELLWLACVSLTQFVHER

115483947_Strep_Oryza_sativa_Japonica_Group__CDC45 LLILPSA--ADADALCALKVLTHVLSLCLLLINWGAHRDTAFVVDSHRPFHGKPSGCLMYELAHTNELLWLACVSLTQFVHER

74096181_Metaz_Ciona_intestinalis__CDC45 VLVMVA---QDVDGLCACKILQSLFKKHMVLLNCGGSIDMIYVIDSRRPYYGTSAALILYELAWDITMLWWAIIGVTQYQNKK

115657931_Metaz_Strongylocentrotus_purpuratus__CDC45 VLVLVA---DDVDAICACKILQTLLQKYVVLINCGGTVNVFFICDSHRPYYGSSAAVVMYNLAWTNDLLWWAIVGLTQLIHKK

213515046_Metaz_Salmo_salar__CDC45 VALLVS---SDIDALCACKILQALFHRYFVLINCGANVDIFFICDTHRPYHGTSAAMVIFELAWTKDMLWWAIIGLTQWVHDK

4502713_Metaz_Homo_sapiens__CDC45 VLLFVA---SDVDALCACKILQALFQHYFILINCGANVDIFFVCDTHRPYHGTSSAMVMFELAWLNDMLWWAIVGLTQWVQDK

189238662_Metaz_Tribolium_castaneum__CDC45 ILLMVH---YDIDSICACKILQNLLKKYFVLINCGGTVNIFFIIDSHRPYYNKASAIAMFKLAWDKDLLWLAIVALTQYILGK

18543207_Metaz_Drosophila_melanogaster__CDC45 ILIVVN---YDIDAICASRILQALFKKYVVLVNCGGCVDTFFICDSHRPYYGRSAALMVFELAWNMDLLWWAIVGITQLLLGK

6323132_Fungi_Saccharomyces_cerevisiae_S288c__CDC45 LVIFVSC--LNIDALCATKMLSLLFKNSLLLVGFGGVIDDIYVLDAHRPTVVNSISAQIYSLLSNLSNLWLNILGTT---LDI

66827851_Mycet_Dictyostelium_discoideum_AX4__CDC45 VLILVA---RDCDSIAACKILTEILKKSIIMINCGGNIDVAYIIDSHRPYYGKSAAVSMYSLSTLDDLLWYAVLGLTQFIHEK

19115605_Fungi_Schizosaccharomyces_pombe_972h__CDC45 VQLFVA---LDPDALCACKLLSTLLKKFIILLNCGTMVDSIYVIDSHRPWYGESITNILFAVASDNDMLWLAIVGLTLEIHCQ

134111787_Fungi_Cryptococcus_neoformans_var_neoformans_B3501A__CDC45 VVILAG---VDVDGLLGARILCSLFKHTLILLSLGSLLTHLHVIDSHRPYYGQSVALTIYLLATDNDILWYSILGVTQYITSH

11498935_Archa_Archaeoglobus_fulgidus_DSM_4304__arCOG000429 PVIIRHH--WDADGTCGGVALEKALTPLVVLVDNGSGLEDVITIDHHFPDSNYTSGVLCVEIARDLDMKHLAAISVVGDRAEG

76802252_Halob_Natronomonas_pharaonis_DSM_2160__arCOG000429 PIRLRHH--ADGDGMCASLPVQIALEPLLFMVDNGSTEEPVLVVDHHHPDYRITTGMMCVELARTEELEHVPAVAGLSDRSKA

110667540_Halob_Haloquadratum_walsbyi_DSM_16790__arCOG000429 PIRIRHH--ADGDGMCASVPVQLALEPFLLMLDNGSTEEPTAVIDHHHPDYRITTGMLCVELARTDEIQHVPAVAGLADRSKA

116754783_Metha_Methanosaeta_thermophila_PT__arCOG000429 PIVIRHH--ADADGISAAVAIETAILPLVVLIDNGSTEEEMVVVDHHHPDFGITTGMLGVEIARENKIKHLAAVSAVGDRSEA

73670910_Metha_Methanosarcina_barkeri_str_Fusaro__arCOG000429 PIILRHH--ADADGITSAIAIERAILPLIILVDNGSTEEDMLVIDHHHPDFGVTAGMLCAEVARSDTIKHLPAVSAVGDRSEA

21228136_Metha_Methanosarcina_mazei_Go1__arCOG000429 PILLRHH--ADADGITSAIAIERAILPLVIQVDNGSTEEDMLVIDHHHPDFGVTAGMLCAEVARSDTIKHLPAVSAVGDRSEA

110622123_envir_uncultured_methanogenic_archaeon_RCI__arCOG000429 PIIIRHH--ADADGICAGVAIERACLPLIVLMDNGSTEEDLLVVDHHHPDFGITAGMLGTELARTEEIKHLAAVAAVGDRSEA

88602981_Metha_Methanospirillum_hungatei_JF1__arCOG000429 PILLRHH--ADADGICSAVAIEQAVVPLIMLTDNGSTEEPMIVFDHHHPDYGITAGMLGTEIARDHQVRHIAAVAGVADRSEA

Maximum likelihood (ML) phylogenetic tree for DHH domain was constructed using the MOLPHY program [[1](#_ENREF_1)] with the JTT substitution matrix to perform local rearrangement of an original Fitch tree [[3](#_ENREF_3)]. The MOLPHY program was also used to compute RELL bootstrap values. Bootstrap values are shown for major internal branches. Designations are the same as for the alignment above.

Maximum likelihood (ML) phylogenetic tree for arCOG00427 was constructed using the MOLPHY program [[1](#_ENREF_1)] with the JTT substitution matrix to perform local rearrangement of an original Fitch tree [[3](#_ENREF_3)]. The MOLPHY program was also used to compute RELL bootstrap values. Bootstrap values are shown for major internal branches. Designations are the same as for the alignment above.

1. Adachi J, Hasegawa M: **MOLPHY: Programs for molecular phylogenetics.** In: *Computer Science Monographs 27;.* Tokyo: Institute of Statistical Mathematics; 1992.

2. Edgar RC: **MUSCLE: multiple sequence alignment with high accuracy and high throughput**. *Nucleic Acids Res* 2004, **32**(5):1792-1797.

3. Fitch WM, Margoliash E: **Construction of phylogenetic trees**. *Science* 1967, **155**(760):279-284.
