# Supplementary material for: The CMG (CDC45/RecJ, MCM, GINS) complex is a conserved component of the DNA replication system in all archaea and eukaryotes
Source: Biol Direct. 2012 Feb 13;7:7. doi: 10.1186/1745-6150-7-7 (PMC3307487; doi:10.1186/1745-6150-7-7)
Supplement: Additional file 2 — The multiple alignment and secondary structure prediction for CDC45 family. The provided data presents multiple alignment, secondary structure prediction and map of subdomains for CDC45 family. [file 1745-6150-7-7-S2.DOCX]

**The multiple alignment and secondary structure prediction for CDC45 family.**

The MUSCLE program [[1](#_ENREF_1)] was used for construction of the multiple sequence alignment. The sequences are denoted by their GI numbers and complete species name. The Jpred [[2](#_ENREF_2)] secondary structure prediction is shown underneath the multiple alignment; “H” indicates positions for α-helices and “E” - β-strands. The four motifs proposed by Aravind and Koonin [[3](#_ENREF_3)] are designated as red boxes. The putative distinct structural regions are shown as follows: regions Cyan – DHH catalytic domain; green – DHH alpha helical region; magenta – potential connector helix; yellow – DHHA1 domain. For DHHA1 domain alignment with the corresponding domain of Sh1221 protein from *Staphylococcus haemolyticus* (PDB: 3dev), COG0618, is shown.

159476804 Chlamydomonas_reinhardtii --------MIVEAQYLLQVY-GAIKN----DCRGSEDQAVMCFAACTDADSVCASQQLMNLFNREGIFFTLIPVDSYEEVKAHCEP-

302829318 Volvox_carteri_f-_nagariensis --------MIVPGQHLRKVY-EAIKK----DSNGSEDQSVLCLAASVDADSVCASQLLLNLFNRESIHFSLVPVACY----------

167523405 Monosiga_brevicollis_MX1 --------MFVSVSGFAGAY-DRICR-----DALQPDKKVLIVAA-MDVDAACACGILTRLFEQDGVQYQVYPVRNYIDVNDISQL-

145526286 Paramecium_tetraurelia_strain_d4-2 --------MKILLNNIKECF-QKILK-----RSEFGKTSVCIIVS-LDVDAICALRILTSLLNRENLQFRMIPVSGYSEMTEAIQK-

146185831 Tetrahymena_thermophila --------MLIDQTSIIHCY-EKIQK-----KAINGCVTVFIFVA-FDADSLCSLKILTGLLKSDNIAYKVLPVTGFSQLEASIDE-

145345736 Ostreococcus_lucimarinus_CCE9901 --------MLVG--ANNFHFAYSTIR------DEGANCPVLVYVSTADVDALCAFRTLKLMLRSDNVAYSVFPVSGYGELQRLGEE-

168017943 Physcomitrella_patens_subsp-_patens --------MVRE--TNPGAF-YDKLR---KAVATSVGSPLLIFPSASDVDSLCALKIVTNLLVQDSVRYSVYPVAGFQSIQSLAGR-

302815970 Selaginella_moellendorffii --------MVRE--SSGAHF-YRRLK---SAASAASSCPLHILPSTVDADSVCALKIFRSILNADNVRHSVFPVSRMADVGSRAAA-

15230255 Arabidopsis_thaliana --------MVRI--KKVESF-YAKLR--ESATSLSSQNPLLIFPSTSDVDSLCALKVITHILESDSIQYSCFPVSSFLEIHKYAGPA

115483947 Oryza_sativa_Japonica_Group --------MVRE--LRLDSF-YARLRAAAAASAADASSPLLILPSAADADALCALKVLTHVLSADSIRFSIYPVASAAAAASLLAS-

74096181 Ciona_intestinalis --------MLIT--DPVKDF-FQKI----------IEERVLVMVA-QDVDGLCACKILQSLFKCDHVQYTLVPVEDKQDLVTEFDS-

115657931 Strongylocentrotus_purpuratus --------MFIT--DIRTQF-YDVI----------LHHRVLVLVA-DDVDAICACKILQTLLQWDHVQYTLVPVSGKDDVEQAFLE-

213515046 Salmo_salar --------MFIT--DIRKEF-YDVV----------VNQRVALLVS-SDIDALCACKILQALFHCDQVQYTLVPVTGWQDLGTAFLE-

4502713 Homo_sapiens --------MFVS--DFRKEF-YEVV----------QSQRVLLFVA-SDVDALCACKILQALFQCDHVQYTLVPVSGWQELETAFLE-

189238662 Tribolium_castaneum --------MYVE--NLKLDF-YEFL----------TGKRILLMVH-YDIDSICACKILQNLLKYKEILYTLAVIRGVEDLKRSYRE-

18543207 Drosophila_melanogaster --------MFVQ--DLRNDF-YRQL----------VGKRILIVVN-YDIDAICASRILQALFKYDHMLYTVVPIMGVTGLKRAYGE-

6323132 Saccharomyces_cerevisiae_S288c --------MYYGISQFSEAY-NKILR----NSSSHSSCQLVIFVSCLNIDALCATKMLSLLFKKQLVQSQIVPIFGYSELRRHYSQ-

66827851 Dictyostelium_discoideum_AX4 --------MVLTTDTKFSEI-YDIIK-----GDSVQGESVLILVA-RDCDSIAACKILTEILKSDLIAYNIKAVSGYEDLENVNST-

19115605 Schizosaccharomyces_pombe_972h- --------MFIKRSDYASAY-LKIKE-----ASVSGGCTVQLFVA-LDPDALCACKLLSTLLKGDFISHKIRPVSGYRDLEQANKT-

134111787 Cryptococcus_neoformansB-3501A MPVVQPPADDLRPSDLTYIHAYNSIVSRVRRTAGAASGGVVILAG-VDVDGLLGARILCSLFKNDDIPYRLIPVGGLTELEEKSDE-

Jnet 15230255 (Jpred sec. str. prediction) ----------------HHHH-HHHHH--HHHH-------EEEEEE---HHHHHHHHHHHHHHH----EEEEEEE--HHHHHHHHHHH

3devA structure ---------------------------------------------------------------------------------------

3devA ---------------------------------------------------------------------------------------

Motif I

159476804 Chlamydomonas_reinhardtii --LVQ-AEEVKT-VVLINCGATDDIRSLC-------ELPDNV----------------RIVIIDHHRPVWHGHNVE----------D

302829318 Volvox_carteri_f-_nagariensis ------EEEVKT-VLLLNCGATEDVRSLC-------NLPSNV----------------RIVVLDHHRPVWHGHNND----------D

167523405 Monosiga_brevicollis_MX1 --LAGGDGQLFCAAILLNVGASYDAAELM--QAHETQPNEHM----------------TIYMFEAHRP-INLYNVF-----------

145526286 Paramecium_tetraurelia_strain_d4-2 --C---GNMYKS-IILLNCGNVYDMSDFI---------KGDI----------------KFFIFDSHKP-INHNNVN-----------

146185831 Tetrahymena_thermophila --CQT-SESIQS-MIFINCGGVLDLTQKWFV-----TEKTKI----------------KAYIFDNHRP-IHHHNIA-----------

145345736 Ostreococcus_lucimarinus_CCE9901 --IPD-DGQQRA-IVLINCGGTEDVKTVM-------GLREGA----------------RAYVFDSHRP-LDLENTR-----------

168017943 Physcomitrella_patens_subsp-_patens --SLRSEDDSIV-VFLINWGASRNLLQLL-------QLGSKA----------------QVFVVDSHRP-IHLHNLS----------D

302815970 Selaginella_moellendorffii --IQ--AEEPVV-VLLINWGGTQDLRRQL-------KLGPRV----------------LVFVVDSHRP-IHLNNWS----------D

15230255 Arabidopsis_thaliana GLCSTSLESPPVTILLINWGCHRDLKLVL-------KLGPSA----------------RVFVVDSHRP-IHLHNLS----------D

115483947 Oryza_sativa_Japonica_Group --FS--ASHPLC-LLLINWGAHRDLRAVL-------P--PAA----------------TAFVVDSHRP-IHLHNLS----------A

74096181 Ciona_intestinalis --H---KEQYKH-MVLLNCGGSIDLLEIL-------EPDDDV----------------MIYVIDSRRP-IDLVNFY-----------

115657931 Strongylocentrotus_purpuratus --H---SEQLKY-VVLINCGGTVNLLESL-------QPDENV----------------VFFICDSHRP-LHLWNIY-----------

213515046 Salmo_salar --H---KEQFRY-FVLINCGANVDLLETL-------QPEEDS----------------IFFICDTHRP-VDVVNVY-----------

4502713 Homo_sapiens --H---KEQFHY-FILINCGANVDLLDIL-------QPDEDT----------------IFFVCDTHRP-VNVVNVY-----------

189238662 Tribolium_castaneum --N---SNDVKY-FVLINCGGTVNLVDLF-------EPEEDA----------------IFFIIDSHRP-THLDNIY-----------

18543207 Drosophila_melanogaster --H---QGDVKY-VVLVNCGGCVDIVELL-------QPSDDV----------------TFFICDSHRP-LDVCNIY-----------

6323132 Saccharomyces_cerevisiae_S288c --L---DDNINS-LLLVGFGGVIDLEAFL-------EIDPQEYVIDTDEKSGEQSFRRDIYVLDAHRP-WNLDNIF-----------

66827851 Dictyostelium_discoideum_AX4 --LLE-NEEIKS-IIMINCGGNIDITNVF------TNLNDQQ----------------VAYIIDSHRP-YSINNIT-----------

19115605 Schizosaccharomyces_pombe_972h- --LLEQNEDIKF-IILLNCGTMVDLNNYL-------VSMEDV----------------SIYVIDSHRP-HNLNNIY-----------

134111787 Cryptococcus_neoformansB-3501A --ALA-SEEIHT-LILLSLGSLLTLTDFF-------NLPKKV----------------HLHVIDSHRP-WNLGNLFDIDLDDEDDED

Jnet 15230255 (Jpred sec. str. prediction) -----------EEEEEE------------------------E----------------EEEEEE--------EEEE-----------

3devA structure ---------------------------------------------------------------------------------------

3devA ---------------------------------------------------------------------------------------

Motif II Motif III

159476804 Chlamydomonas_reinhardtii DTNTLVLVDED--DPVPKASVPVYNPNEQEEDEEDSGSEASEAEKDDEEDQEAGVSGDGDEDE----------DDLGVRPGRKRR--

302829318 Volvox_carteri_f-_nagariensis DMDTLVLVDDD--DPVPKPAVPSYDQQLDNEVAQSDDSASESDGSDLDDVNDDDNEEGVGSDG--------------EQTRKRHR--

167523405 Monosiga_brevicollis_MX1 --GDVQIFDDG--ETSTA--IPERDAVYLT-------------DDEDEDDEDDRSDASDNEDT---------ASEGGTPEKRTKR--

145526286 Paramecium_tetraurelia_strain_d4-2 NEKSIYIIDDG--MGQLEKCPEE--------------------EIKEMSDGDEDSIDEDDSGS------------HSSQIQRKQQ--

146185831 Tetrahymena_thermophila DKNNIVIIDDG--TQNQQNCPEREDEILQMTAQHEEDEDISDNDNEDQDYSEEDDDDLQEYEDEDEGFDENGENKLKNKLDKKRKDL

145345736 Ostreococcus_lucimarinus_CCE9901 ADNQDVLVMRD--DKEGEESFPE-------------------PDSEDDSDSDDDDDEDDDAGA------------KGGESPRTRR--

168017943 Physcomitrella_patens_subsp-_patens LNPQVTILFTH--DDETQSDTPYGFPLYRL--SSFISTNDSNLDVYEDSESDLDSDSDSDDDG---------------RRRKRRK--

302815970 Selaginella_moellendorffii HNQQVTVLYTR--EEEMERDIVYDFDVGSLADFSFMTAASDGEEDEDEEESDDEEELDGGGGG---------DKHDNMRKRGRGR--

15230255 Arabidopsis_thaliana YNEQVVVLHTD--DDERQGDLAYDFDVLKLANESFQLRVEDAGEESDEEEEDEEEDEEDDDDD------------DGDRPSKRRK--

115483947 Oryza_sativa_Japonica_Group ANDRVVVLFTT--DDEHTADLSYDFDVSSLADASDLSAQGEADDHLRVAEEDEDSDASDSDSD------------GEGGRRKRRR--

74096181 Ciona_intestinalis CERQVYLILKQ--NQEEAQLIPDYDHIYRN--------------------YDSDNEDSDTEYE---------------QSSKRHK--

115657931 Strongylocentrotus_purpuratus NETQIKLLMSP--DDDFD--IPSYEEVFRD------------------DESDSDDSGAESDSS--------------EPTGKRRR--

213515046 Salmo_salar NDTQVKLMIKQ--DDDLG--VPSYDDIFRD-----------------EEEEGGDDSGNESDGG-------------SEPSGKRRR--

4502713 Homo_sapiens NDTQIKLLIKQ--DDDLE--VPAYEDIFRD------------------EEEDEEHSGNDSDGS--------------EPSEKRTR--

189238662 Tribolium_castaneum SDGQVRLLWTS--EEDLE--VPDFHAVYRD---------------DSDEESDEEPESGDEDEG---------------RAAKKRR--

18543207 Drosophila_melanogaster SDRQVCILG----DASLEENIPAFETIFYD---------SEGEDEDEDESSDTEQQHDDSGAG---------ESDQEDQAPRSRK--

6323132 Saccharomyces_cerevisiae_S288c GSQIIQCFDDGTVDDTLGEQKEAYYKLLELDEESGDDELSGDENDNNGGDDEATDADEVTDED----------EEDEDETISNKR--

66827851 Dictyostelium_discoideum_AX4 NESSVLIIDDG--TYIEQDELQQLSDDSEEEEEELEEEEAEEDVVEDEAEEAEEAEEAEEAEEAEFDPENEDNPENDEDDGGSEN--

19115605 Schizosaccharomyces_pombe_972h- IENNIFVFDDG--DIEED--MNKIHDAWYAFNSHELSDEENSDSSNEREEEVEDDNRSVESYS------------SSDYQARSRR--

134111787 Cryptococcus_neoformansB-3501A AHGKVWIWGDG--DEFSEN-MDQLRKSFEALQFLPQKDSDEDSDSDEESEAEEEEPEEEDEDE-----EGDDDAKDEDGWSRKRR--

Jnet 15230255 (Jpred sec. str. prediction) ---EEEEEE-----------------HHHH--------------------------------H------------HHH-HHHHHH--

3devA structure ---------------------------------------------------------------------------------------

3devA ---------------------------------------------------------------------------------------

159476804 Chlamydomonas_reinhardtii ---RRSTGADQEERPAKRSPEARAQARAER----------RKLAELIDAYYSDCNGYGKPSSLLLFALVNTAQHDD-NFHL--WCAI

302829318 Volvox_carteri_f-_nagariensis -------GHDAGVSPPKRGPAARAENVAAR----------RKRAEEVDAYYSDRNGYGKPSSLLLFSLCHELQHDD-NFHV--WCAI

167523405 Monosiga_brevicollis_MX1 ------------------QRYEDRRLRRLQ-----------------TQSYYEYSYHTTPISVLLWHLVMEKRRES-NDSL--WRAM

145526286 Paramecium_tetraurelia_strain_d4-2 ---------------QKLQRSLRKQLIQMH-----------------EDYYSQGTYYSRPSSTVVYTLAQQMNHDC-NDHL--WYAI

146185831 Tetrahymena_thermophila AYEDEEEEEFGKKDRREKNRRKRRSDKKLKEKKMEKKLKIEQHKQKMENYY-EGFYYGKATSMLMYKICQQANKEN-NNYL--WYTI

145345736 Ostreococcus_lucimarinus_CCE9901 --------------------LRKAEKQRER-----------------AAYYARGSFYGRSSGMVMYDIAYRMSKDRLENYLPLWLAV

168017943 Physcomitrella_patens_subsp-_patens ------------KSYNDGDDEQRREEELLK-----------------AEYYRMGCFHGRPSGCLMFDIAHTLHKNT-NELL--WLAG

302815970 Selaginella_moellendorffii ----------GRDDSSDHADNLRRDMRRRK-----------------KDYYSTGTFHGRAAGRQVYDISHALHINT-HELL--WLAC

15230255 Arabidopsis_thaliana -----------------MGDGVKVFKKLKR------------------DYYKMGTFHGKPSGCLLFELSHMLRKNT-NELL--WLAC

115483947 Oryza_sativa_Japonica_Group ----------LSDDAEANGDPERLFGKLRR------------------EYYRLGTFHGKPSGCLMYELAHALRKNT-NELL--WLAC

74096181 Ciona_intestinalis ---------------FDQETLEKKREKRIW---------EEKRKEIIFD-YEEYSYYGTSAALILYELAWKMSKDD-ITML--WWAI

115657931 Strongylocentrotus_purpuratus -----------YDEDALVRKMNRRQERRNW---------DDKRSKILFD-YEEFSYYGSSAAVVMYNLAWKKSKDT-NDLL--WWAI

213515046 Salmo_salar -----------YDEGEVERRIERQRATREW---------EGRRREILFD-YEQYEYHGTSAAMVIFELAWVLTKDT-KDML--WWAI

4502713 Homo_sapiens ------------LEEEIVEQTMRRRQRREW---------EARRRDILFD-YEQYEYHGTSSAMVMFELAWMLSKDL-NDML--WWAI

189238662 Tribolium_castaneum ---------------LNEEAILKRRERRLW---------EAKRFDIIAE-YSQYTYYNKASAIAMFKLAWFLNKDD-KDLL--WLAI

18543207 Drosophila_melanogaster ----------LSRLERHEQRILKQRARRQW---------ESERDRIMFE-YTQFSYYGRSAALMVFELAWKLSKDN-MDLL--WWAI

6323132 Saccharomyces_cerevisiae_S288c -----------GNSSIGPNDLSKRKQRKKQ---------IHEYEGVLEEYYSQGTTVVNSISAQIYSLLSAIGETN-LSNL--WLNI

66827851 Dictyostelium_discoideum_AX4 ----------IDENERKSKKKNKKDKKSKR---------KHRKKKKKKSEKLEKTYYGKSAAVSMYSLSTFLNKQNLDDLL--WYAV

19115605 Schizosaccharomyces_pombe_972h- ----------RFSEETTQRRAEIKEKRKKR----------KEFASILSEYYEKGSWYGESITNILFAVASMLGRED-NDML--WLAI

134111787 Cryptococcus_neoformansB-3501A ----------RE-DSVAARRKRRRDDDRPR--KLPKAIK-EAHQERIAKYYNHGTYYGQSVALTIYLLATVLERAD-NDIL--WYSI

Jnet 15230255 (Jpred sec. str. prediction) -----------------HHHHHHHHHHHHH------------------HHHHH------HHHHHHHHHHHHH-----HHHH--HHHH

3devA structure ---------------------------------------------------------------------------------------

3devA ---------------------------------------------------------------------------------------

159476804 Chlamydomonas_reinhardtii VGLTEQLLFHQISKQQYNTWREGLEQKTHVQADPQEE---LDNEAANGLLHIPRQK-----------------VHVE---PFDDLRL

302829318 Volvox_carteri_f-_nagariensis VALTEQLLFQQISKQQYNTWRDKLAVHVTMHHDQDQE---DAAQDGISSLLSMPRHK----------------VHVE---ACEDLRL

167523405 Monosiga_brevicollis_MX1 VALVEAFVEDRISAELYSRMYSDLKRDMERLNASDES---EGIV-----------------------------RTVHLREIKQDFRF

145526286 Paramecium_tetraurelia_strain_d4-2 LGLTDAYIHMRLNQKNYDLIFEELQNEVIRLNIHFEE---TQGDA----------------------------KKIGGVFIENEYKF

146185831 Tetrahymena_thermophila LGATDLLIHGKITQQQYDQMYEELVIEVEKVNIQKQE---FSQDLNESIANEQRQ------------------KNIG---FIEASN-

145345736 Ostreococcus_lucimarinus_CCE9901 LSMTDQYLHQRLSHEMYTSCVMELATRVSAMSNADMP---TTRSLEDGTIVKAFDE-----------------RRLQ---YNEEFRF

168017943 Physcomitrella_patens_subsp-_patens VALTDQFVHERLSKERYDSTQSELEGFVALAGNFESS---TSVTLKDGTRVRAPDV-----------------SRII---FDEEPRL

302815970 Selaginella_moellendorffii VSLTDQFVHERLTMERYQSGVMELDTYVKTSGNSEMV---TKVTMKDGTVVKAPDR-----------------SRIT---NDEEPRL

15230255 Arabidopsis_thaliana VSLTDQFVHERLTDERYQAAVMELEQHINSSGNIDKI---TSVTLKDGTKVRAPDC-----------------SRIS---YEEEPRL

115483947 Oryza_sativa_Japonica_Group VSLTDQFVHERITNERYQAAVMELEQHINGSGNLDPSGVGAVVTLKDGTKIRAPEA-----------------SRIA---YEDEPRL

74096181 Ciona_intestinalis IGVTSQYQNKKIGREKYISSVLELQGHLSRLNHREED---NENTTSIDS------------------------IKIN---FEHDLDI

115657931 Strongylocentrotus_purpuratus VGLTDQLIHKKIDREKYVSDVTELNRHVARHNHRGED---EENTVSVNT------------------------MKIS---FLPDLQL

213515046 Salmo_salar IGLTDQWVHDKITHMKYVTDIATLQRHVSRHNHRNED---EDNSLSIDC------------------------MRIS---FEYDLRL

4502713 Homo_sapiens VGLTDQWVQDKITQMKYVTDVGVLQRHVSRHNHRNED---EENTLSVDC------------------------TRIS---FEYDLRL

189238662 Tribolium_castaneum VALTEQYILGKIENSQYVLAVGELQSHSNRLRNRSND---TDGLTS---------------------------LRIT---YENDLKL

18543207 Drosophila_melanogaster VGITEQLLLGKIESGAYTLELEQIQSHVSRLTNKTND----QNTMSA--------------------------SKIT---FENDLHL

6323132 Saccharomyces_cerevisiae_S288c LGTTS---LDIAYAQVYNRLYPLLQDEVKRLTPSSRN------------SVKTPDT-----------------LTLN---IQPDYYL

66827851 Dictyostelium_discoideum_AX4 LGLTDQFIHEKITLDSYEQQYKNFKELILNNYPTDGD---DDQEERYRPGNDEDDYDENGDLKRHPSSMFLHGDKII---PSDDFRF

19115605 Schizosaccharomyces_pombe_972h- VGLTCLEIHCQSSKKYFNRSYSLLKDEVNRLNPSPLE-----NQIVGRAHGKTPHD-----------------QSIR---LEDEFRF

134111787 Cryptococcus_neoformansB-3501A LGVTHQYITSHIDREKYEEYHAIFLDEVVRLNHEPDP---------VALRTPNPDN-----------------RNIS---KSEELRF

Jnet 15230255 (Jpred sec. str. prediction) HHHHHHHHHH---HHHHHHHHHHHHHHHH---------------------E-----------------------EEE----------

3devA structure ---------------------------------------------------------------------------------------

3devA ---------------------------------------------------------------------------------------

159476804 Chlamydomonas_reinhardtii TLLRYWTIEDSLRYTGYVAARLQTWRQKGLDNLRSLLTYMCIPLKHATTAY-KGLREVYNSQLRAQLPRFAPQHMLAWDSLHLSSFR

302829318 Volvox_carteri_f-_nagariensis TLLRYWTIEDSLRYTCYVAARLQTWRQKGLDNLRSLLTYMCIPLKHASTAY-KGLREVYNGQLRSQLPRFAPQHMLAWDSLHFNSFR

167523405 Monosiga_brevicollis_MX1 PLYRHWNIYDAMCNSRYVAARVDVWKTKGEAALCNMLAQVGVSKQTCKQNFIAWTDGNARKRVMESLIQALTDMGI--DHCTIPSFE

145526286 Paramecium_tetraurelia_strain_d4-2 LLMRQQSLYNSMYYSSYVGTKLKLWKDRDNKRLEEFMAKLGIPLNEAKQDF-RYMNQKYKQILKSQIAEVASKFQM--DDILYRSFV

146185831 Tetrahymena_thermophila ---EQYLVSQSIYYSNYFATKLEIWNENGDQKITRLIALLGIPLEEAKQQY-KFMKAQFKEKLKDNIHSVATKYKL--YNVLFNSFV

145345736 Ostreococcus_lucimarinus_CCE9901 MLLRHWTLYESMLHSNYVATQLQTWTERGRANLNSLFAHVGIPLDVAKQKY-SHMSPAQVKQFEERLDEYGASHGL--DNVKFWSFQ

168017943 Physcomitrella_patens_subsp-_patens MLLREWSLYEAMMCSFYVAPKLKTWGDSGLKTMKLLIAKMGISLAECQQQY-QYMKNETRKGLKQMLEKVGKDAGL--GDLYFRNLY

302815970 Selaginella_moellendorffii MLLREWTLYDSMLCSSYVATKLKTWSDGGLKRLKLLLATMGIALVEGQQKY-EYMSGETRRRMKEEFQRSSPQFGL--TDLYYRSFQ

15230255 Arabidopsis_thaliana MLLREWTLFDSMLCSSYIATKLKTWSDNGIKKLKLLLARMGFALIECQQKF-PYMSLEVKRKMKQEFDRFLPEYGL--NDFYYRSFL

115483947 Oryza_sativa_Japonica_Group MLLREWSLFDSMLCSSYVATKLKTWSDNGLKKLKLLLARMGFPLADCQKRF-QYMSMEVKRKMRDEFDRFLPEYGL--TEFYYRSFL

74096181 Ciona_intestinalis ALYRHWSLFESLCHSLNTGTTFKVWTNLGMKRLHQFLAEMGIPLTQCKQSF-AFMETKTRDNLQTLVQESAEKFGL--RNIKFHSFT

115657931 Strongylocentrotus_purpuratus VLYRHWSIFESLKHSSYTACNLKLWTLKGQKKLNEFLADMGLPLAQCQQKF-SAMDITYKNSIRELLESSARKFGL--ENITLPSFL

213515046 Salmo_salar ALYQHWSLFESICNSSYTSCNFKLWSMHGQKKLQEFLADMGLPLKQVKQKF-NSMDMSVKENLREVIEESSNKYGM--KDIRIQTFG

4502713 Homo_sapiens VLYQHWSLHDSLCNTSYTAARFKLWSVHGQKRLQEFLADMGLPLKQVKQKF-QAMDISLKENLREMIEESANKFGM--KDMRVQTFS

189238662 Tribolium_castaneum VLYRHWSVESSLKYSMFTACKLKLWSHRGSKKLYELLADMGLPLSQSQQAF-ESMDLQLRKEFHQSIEKLSEKYNL--EDLVFTSFV

18543207 Drosophila_melanogaster VLYRHWPVTESMRYSRYSSCQLKLWTLRGEKRLHELLLEMGLPLVHARQTY-GAMDLVLRKEFFSMVERLAEKYDI--ADIVYGTFT

6323132 Saccharomyces_cerevisiae_S288c FLLRHSSLYDSFYYSNYVNAKLSLWNENGKKRLHKMFARMGIPLSTAQETW-LYMDHSIKRELGIIFDKNLDRYGL--QDIIRDGFV

66827851 Dictyostelium_discoideum_AX4 MLYRHWNLYESLFNSRYVACKLRVWKAKGRFQLESLFAMMGIPLDQVKQKY-NSMNVHYKKQLKQLLAINGPKFGL--VNLYFNSFL

19115605 Schizosaccharomyces_pombe_972h- MLVRHWSLYDSMLHSAYVGSRLHIWSEEGRKRLHKLLAKMGLSLVECKQTY-IHMNMDLKKTLKSSLKRFAPFYGL--DDVIFHSFT

134111787 Cryptococcus_neoformansB-3501A MLFRHWDLYNAMLHSGYVAGRLGIWKEKGRSKLRGLLAKMGYSIQQCNQAW-SHMDMELKRQLPEVLERVGPEYGL--VELSYPSFT

Jnet 15230255 (Jpred sec. str. prediction) ----HHHHHHHHH------------HHHHHHHHHHHHHH----HHHHHH--------HHHHHHHHHHHHHH---------EEE--EE

3devA structure ---------------------------------------------------------------------------------------

3devA ---------------------------------------------------------------------------------------

159476804 Chlamydomonas_reinhardtii LLYKHE--EISASDMVFALLGLLTDAK-----------------------PKEKSW-------------------------------

302829318 Volvox_carteri_f-_nagariensis LLYKHE--ELGASDMVFALLGLLTEA------------------------EPGNPD-------------------------------

167523405 Monosiga_brevicollis_MX1 YQFEYQ-RALSASDLAYAVGAQFLFP------------------------RAEGDW-------------------------------

145526286 Paramecium_tetraurelia_strain_d4-2 RQSDTK-IQIAASDMVYAVNAILEYPQALLSKQLMQ--------------HQCSEV-------------------------------

146185831 Tetrahymena_thermophila RQIDEK-TQVSSTDMVYCLTAILECPKQILQNIFEDPLKVGQSIQTDKTSDNSEEG-------------------------------

145345736 Ostreococcus_lucimarinus_CCE9901 YSHGYA-LRVCAADVVYGATALLEGM--LDRE------------------EEDRTP-------------------------------

168017943 Physcomitrella_patens_subsp-_patens RFHGYN-DQVSAADVVYGVTALLEASTT----------------------DMKKDW-------------------------------

302815970 Selaginella_moellendorffii KLHGYS-SEVSAADVVYGLTALLEASC-----------------------DDHGGQ-------------------------------

15230255 Arabidopsis_thaliana RLHGYS-SRVSAADVVYGITALLESFLG----------------------SGGSSA-------------------------------

115483947 Oryza_sativa_Japonica_Group RVHGYR-SKVSAADVVYGVTALLESLNAESKD------------------SKGSSA-------------------------------

74096181 Ciona_intestinalis AQCGFN-HKLCASDVVFSVDSILTNT------------------------ESSKPE-------------------------------

115657931 Strongylocentrotus_purpuratus AQYGFK-NKFCAFDIAYAVEAILESV------------------------DKNKSS-------------------------------

213515046 Salmo_salar VHFGFK-NRFLASDVVHAAAALLEST------------------------EKDDSP-------------------------------

4502713 Homo_sapiens IHFGFK-HKFLASDVVFATMSLMESP------------------------EKDGSG-------------------------------

189238662 Tribolium_castaneum LQYGYR-NKYCASDIVYAMFAILESS------------------------PKETPE-------------------------------

18543207 Drosophila_melanogaster LSYGYR-SRYAAADYVYALLAIMESV------------------------KKHKTP-------------------------------

6323132 Saccharomyces_cerevisiae_S288c RTLGYR-GSISASEFVEALTALLEVGNSTDKDSVKINNDNNDDTDGE---EEEDNS-------------------------------

66827851 Dictyostelium_discoideum_AX4 KKYSNN-SEISASDTVYAVTALMESDNLESDQ------------------SDQDIW-------------------------------

19115605 Schizosaccharomyces_pombe_972h- RTYGFK-CTLSASDVSYAISALLEMGNTGVLLQSKTVARSPDMTEEEYL-EKFENA-------------------------------

134111787 Cryptococcus_neoformansB-3501A RAYGFQLSSLSAADAVEVISSLLDIAVGVRLEVDREGG------------KGGGEWFGGTTRWSVGTREAEMGIAASEPGERGEGIE

Jnet 15230255 (Jpred sec. str. prediction) EEE-----EEEHHHHHHHHHHHHH---------------------------------------------------------------

3devA structure ---------------------------------------------------------------------------------------

3devA ---------------------------------------------------------------------------------------

159476804 Chlamydomonas_reinhardtii -H-----------RAAFNDASNALHVQRNLNVVEKGIELAKRMCSDVVHECGLMITS-GKVKGGRNADYRWVNVAE-SNGLANPRFT

302829318 Volvox_carteri_f-_nagariensis -SQR----------TAFNNAQSALHVQRNLHIVERGVELAKQMCQDVVHECGLMITA-GKVKGGRSADYRWVNVAD-SNALANPRFR

167523405 Monosiga_brevicollis_MX1 -Q------------QAFYTSLDILT-MRKRAAFEDGCNKAMELERVLLRQVQTCLNN-GGTSMAKR--YRLYSILD---TSDHKYFH

145526286 Paramecium_tetraurelia_strain_d4-2 -IEKLKTSETAAKFQNFFCALDTLG-SKSDDLFQQGVQMALEFQAALVDEANNLIEN-NELYPCKH--FRYGILKN-ESPQQKKFFQ

146185831 Tetrahymena_thermophila -SKNYNEKDIFDRIKNFWWAYETIS-SKEAVLILKGIDIAIQFQKAMVNEAKFVIDR-DLITQCSD--FRFLKLNN-DTYSQNQYFQ

145345736 Ostreococcus_lucimarinus_CCE9901 -A------------DNFWRASQALS-LGNWDEMEAGISHAIRLQQAVMRQGGIALASSAHIRTTGS--LRCFSLLDHGSPADVKLFM

168017943 Physcomitrella_patens_subsp-_patens -T------------DNFCTALEALS-PDKWDHLRAGMQLAIKVQRACMSIGSAAIVKKGEIKMCKA--FRYFELPK---CVDAELLA

302815970 Selaginella_moellendorffii ----------------FMRAYSALA-PKKWHELQQGMELAIKVQRAILRQGSLAVTKQGFVKNTKM--FRWFKLED---GHDVELLA

15230255 Arabidopsis_thaliana -S------------KQFGEAYDALS-LNNLDKLRSGMQQAIKVQRAILRQGSAAITKSGCIRSGRK--FRWVKIED---SMDAKYLG

115483947 Oryza_sativa_Japonica_Group -A------------EQFWVAYSALS-LSNVDQLRKGMQSAIEIQRAILRQGSSAITKTGFIRSAKK--FRWVKLDD---PVDTDKLC

74096181 Ciona_intestinalis -SYED--------NSNFVDALEGLS-RITSPKLEEGLQLAKVQLRAIKNNVGSFIDI-GQVLSYGP--FLYTNIKE--GSPDCTLFA

115657931 Strongylocentrotus_purpuratus -E------------ERFLDALDCLS-RSYIDKLHKGLDASKLQLQAVVIQVQSLLDM-GQVVSAGP--FLYAFLPE--GTPETKFFA

213515046 Salmo_salar -G------------DNFIKALDCLS-RSNLERLHGGIDLAKKKLMAIQQTVASCICT-NLILSQGP--FLYCYLLE--GTPDVKLFS

4502713 Homo_sapiens -T------------DHFIQALDSLS-RSNLDKLYHGLELAKKQLRATQQTIASCLCT-NLVISQGP--FLYCSLME--GTPDVMLFS

189238662 Tribolium_castaneum --------------ECFNSALDCLT-RNKKDVVQKAIERAKIITKTIFKTVQAAIDM-KQIITAGN--FVYYIIQE----VNWYMFS

18543207 Drosophila_melanogaster -E------------DCFLEASDALS-RQHKQLLSAGIDQAKLLHAAVFRQVQSSLEA-RQVHSAGS--FFYYVLQE-----EHAFFS

6323132 Saccharomyces_cerevisiae_S288c -AQKLTNLRKRW-VSNFWLSWDALD-DRKVELLNRGIQLAQDLQRAIFNTGVAILEK-KLIKHLRI--YRLCVLQD---GPDLDLYR

66827851 Dictyostelium_discoideum_AX4 -E------------QNFWEAYDSIS-NKNIDLLKIGLKQSIQLQKEITRQVTSMIEK-RSVILSGP--FRYAFITE---SSDLKYFI

19115605 Schizosaccharomyces_pombe_972h- -QNQE------W-LHNFYDAYDALD---DVDSLERALKLAMHLQRAIVRTGITLLEK-RAIKTLRS--FRFGLINE---GPDLKIFM

134111787 Cryptococcus_neoformansB-3501A GEESEEKKDQDWHVTNFWIAYDACD---DISLLRRSLPLAMALHRAIIRAGSDLLDK-SIIRTLRN--FRLTILSE---GPDLRLFC

Jnet 15230255 (Jpred sec. str. prediction) --------------HHHHHHHH-------HHHHHHHHHHHHHHHHHHHHHHHHHH-------------EEEEEE---------HHH-

3devA structure ------------------------------------------HHHHHHHHHHHH--------------EEEEEE-------Hhhhhh

3devA ----------------------------------------KDPKLXPFQGYVLQ----NFELsdSHE-YCQIKI------TNdvlkq

159476804 Chlamydomonas_reinhardtii HPAVLKYMALFLRDATSHR-----------YSSNDARRPMVVAG-PPDEGGMCCVVAVHAKH--------ISGNKLQKNPFARPFIE

302829318 Volvox_carteri_f-_nagariensis HPTVLKYMALFLRDATSCR-----------YSSSDARRPMVVAG-AADERGLCCLVSVHAKH--------ISGNRLQNNPFARPFIE

167523405 Monosiga_brevicollis_MX1 SYGMLHRLARFLVDHRRFS-----------IGARRSAEHHIVAS---RADDKILLVGIWAKKR-------RQEGDAVPNKFGSLFER

145526286 Paramecium_tetraurelia_strain_d4-2 HPQSLQKLALLLMDIYKE------------KGYKQSNKSVILV---NQLGELFMVVGVVGGM--------SF-SGQEKNQFGQYFLR

146185831 Tetrahymena_thermophila HPYSLQKLALFLMGIFKEK-----------QKSNQPIKPIVLSI-KNSITQTQTIIGVVGN---------HY-SASTKNDFAFKFQS

145345736 Ostreococcus_lucimarinus_CCE9901 HPLSLLKLALFVQDAL--------------RVTKKRLRPLVCIGPSAEDESLALIVGVTAKP--------NTDDAAGGNFFTHSFKM

168017943 Physcomitrella_patens_subsp-_patens HPLSLTKLCYFVMDSL--------------KEQGRAVKPMICYGIIPGPANEALIVGVSHRP--------RL-GATNGNRFGLVFRA

302815970 Selaginella_moellendorffii RPMALTKLCYFVMDAL--------------REQGARSKPLVCAI-RCPQSDKALVVGVSHRL--------RL-GAQMGNRFGSVFKT

15230255 Arabidopsis_thaliana YPQALTKFCYFLMDAL--------------REKGARMKPMLCAC-ASQQPGKILVVGVCGKP--------RL-GAVRGNAFGNAFRK

115483947 Oryza_sativa_Japonica_Group QPQALTKFCFFLMDAL--------------RERGARMKPLICAC-LAREPEKVLVVGVCGKP--------RL-GAVKGNAFGNAFRS

74096181 Ciona_intestinalis QPLFLVTFSHFLLHSYMRSLGKS-------KRDRARNLPLVICS-PSPQEGSTMVVGIPPLS-----------EESRKNLFGNAFHH

115657931 Strongylocentrotus_purpuratus HPNCLSMLARFALEAYVKMS----------KSKKAKNLPLVMTAPLNSETGTSLVIGVPPLP--------EL-EQSSRNFFGRAFEQ

213515046 Salmo_salar KPMALTLLCKYLLKAFVRST----------RNKRCKLLPLIIAAPMDVEKRTVIVLGIPPES--------E--TSDKKNFFGRAFEK

4502713 Homo_sapiens RPASLSLLSKHLLKSFVCST----------KNRRCKLLPLVMAAPLSMEHGTVTVVGIPPET--------D--SSDRKNFFGRAFEK

189238662 Tribolium_castaneum NQYILLLLAQFILRAYVSMS----------RNRKAPDLPLIISAPKNLDLGTCVILGIPPLR--------Q---NSPKNNLGRAFEE

18543207 Drosophila_melanogaster YPYALGLLARFLLRGHVATS----------RARQASDLPLIASCPLNASEGMCLLVGIVPVR--------E---DSPRNFFGKAFEQ

6323132 Saccharomyces_cerevisiae_S288c NPLTLLRLGNWLIECCA-------------ESEDKQLLPMVLAS-IDENTDTYLVAGLTPRYPRGLDT--IHTKKPILNNFSMAFQQ

66827851 Dictyostelium_discoideum_AX4 HPLALTKLGLFMMDAF--------------ISMGKAKRPFLIGA-LNENKNSYLIVGISGSH--------S--TDIQSNTFGEYFRK

19115605 Schizosaccharomyces_pombe_972h- HPLALTKMSLWIAEAINEQER---------EFGKLRHLPLVLAA-FVEEKNRYLIVGTSTSAFTSNEDDDDD-DGHGHNRFGVAFQE

134111787 Cryptococcus_neoformansB-3501A HPSPLSRLALWLVDATRDRWVEKIARQNAHSGGKVKSLPFVVAC-LNEEKGTFSVVGVTGAP--------EF-GDVRKNKFGLAFQQ

Jnet 15230255 (Jpred sec. str. prediction) -HHHHHHHHHHHHHHH--------------HH-------EEEEE-E-----EEEEEEE----------------------HHHHHHH

3devA structure ----HHHH---HHH----------------------EEEEEEE-----EEEEEEE-------------------------------H

3devA fdiqPNEASQFVNTVA--------------DIsgLKIWXFGVDe-gdqIRCRIRSKGI--------------------------TIN

159476804 Chlamydomonas_reinhardtii TASALHIFQQK---SAFENTTFHLRKEEVGPFLDRLQHVVKEYTEEAKRLEQVAAA-------------------------------

302829318 Volvox_carteri_f-_nagariensis TASALNIVPLK---SAFENATYHIRKEDVTAFLGKLQDVVADYRAVAQQAQAVQG--------------------------------

167523405 Monosiga_brevicollis_MX1 MTDLHASLYEGTTATQFERPMLELKPDRMQEFCNVLVRVLTESA-------------------------------------------

145526286 Paramecium_tetraurelia_strain_d4-2 KVQELNLQFRQ---DSFETSVIEIHKDDFPNFIDAITDFKKN---------------------------------------------

146185831 Tetrahymena_thermophila AAKALNLQYKQ---DDFETSIIEIKDQDFDVFLDEITLAK-----------------------------------------------

145345736 Ostreococcus_lucimarinus_CCE9901 AAERIHARFRH---DSFEASVIQVARRDLGPFIEALSDIDAERLARLRMAAA-----------------------------------

168017943 Physcomitrella_patens_subsp-_patens IAEKIGSNFSH---DAFESSWIHLPSDDVTRFMQELQSSLASYNSL-----------------------------------------

302815970 Selaginella_moellendorffii VAANLGVDFQQ---DGFDAAWIQTDAASVGAFMTQLSENLPK---------------------------------------------

15230255 Arabidopsis_thaliana AAQESRADYFH---ELFESSWIVLDASAVNSFMIRLTEKL-----------------------------------------------

115483947 Oryza_sativa_Japonica_Group AAEEIGADYFH---DMFESSWIVLDVVAVSSFMIRLTEKL-----------------------------------------------

74096181 Ciona_intestinalis ASERTKSRTQH---HFFNPAIITVVNEDRSKFIDALITIMS----------------------------------------------

115657931 Strongylocentrotus_purpuratus AGKKTGSRTLH---NHFDSSIMELKTGDRSKFFDALISLLS----------------------------------------------

213515046 Salmo_salar AAESTSSRTLH---DHFDTSIIELKMEDRTKFLDALITLLS----------------------------------------------

4502713 Homo_sapiens AAESTSSRMLH---NHFDLSVIELKAEDRSKFLDALISLLS----------------------------------------------

189238662 Tribolium_castaneum AAENINYEVLS---DYFDTSYFEINIKDRTRFFDALTALFDK---------------------------------------------

18543207 Drosophila_melanogaster AAQKSGVALLQ---DFFEPAVVQLRQSDLTRFLDSLTVLLA----------------------------------------------

6323132 Saccharomyces_cerevisiae_S288c ITAETDAKVRI---DNFESSIIEIRREDLSPFLEKLTLSGLL---------------------------------------------

66827851 Dictyostelium_discoideum_AX4 SAEYTDATFKY---QSFDTSIVEVSKTDLHKFVEHLHGTMQTTQISK----------------------------------------

19115605 Schizosaccharomyces_pombe_972h- VANMTSATLQM---DCFEASVIECQKSDLGVFLESLSFKTLL---------------------------------------------

134111787 Cryptococcus_neoformansB-3501A AASFSNATASL---DMFDTSVVEVGREDLQSFIEHLHLHSV----------------------------------------------

Jnet 15230255 (Jpred sec. str. prediction) HHHH---EEEE---------EEEEE-----HHHHHHHH-------------------------------------------------

3devA structure HHHHH---EEE---------EEEEEE--HHHHHHHHHHHHHH---------------------------------------------

3devA DVANQFGGGGH-------PNASGVSVySWDEFEELAQALRQKLLEHH----------------------------------------

1. Edgar RC: **MUSCLE: multiple sequence alignment with high accuracy and high throughput**. *Nucleic Acids Res* 2004, **32**(5):1792-1797.

2. Cuff JA, Clamp ME, Siddiqui AS, Finlay M, Barton GJ: **JPred: a consensus secondary structure prediction server**. *Bioinformatics* 1998, **14**(10):892-893.

3. Aravind L, Koonin EV: **A novel family of predicted phosphoesterases includes Drosophila prune protein and bacterial RecJ exonuclease**. *Trends Biochem Sci* 1998, **23**(1):17-19.
