# Supplementary material for: The CMG (CDC45/RecJ, MCM, GINS) complex is a conserved component of the DNA replication system in all archaea and eukaryotes
Source: Biol Direct. 2012 Feb 13;7:7. doi: 10.1186/1745-6150-7-7 (PMC3307487; doi:10.1186/1745-6150-7-7)
Supplement: Additional file 3 — Results of sequence similarity searches for highly diverged archaeal RecJ homologs. The provided table reports the parameters used for sequence similarity searches and statistical support values. [file 1745-6150-7-7-S3.DOCX]

| Method/Program for sequence similarity search | Query | Hit | Statistical support | Details |
| --- | --- | --- | --- | --- |
| HHpred | Hbut_0430 (arCOG00432) from *Hyperthermus butylicus* | LOAD_CDC45 profile | Probability=98.3%; E-value=0.0001 | Default, all databases used |
| PSI-BLAST | SSO0295 (arCOG5902) from *Sulfolobus solfataricus* | Msm_1193 (arCOG00427) from *Methanobrevibacter smithii* | E-value= 5e-08 | Hit identified after first iteration.  Parameters: NR database (archaeal subset only); inclusion threshold E-value=1e-04; no low complexity filtering; no composition-based statistics |
| PSI-BLAST | MJ_1071 (arCOG00433) from *Methanocaldococcus jannaschii* | region 195-425 aa. to MJ_0977 (arCOG00427) from *Methanocaldococcus jannaschii* | E-value= 4e-19 | Hit identified after first iteration.  Parameters: NR database (archaeal subset only); inclusion threshold E-value=1e-04; no low complexity filtering; no composition-based statistics |
| PSI-BLAST | Msed_1882 (arCOG05902) from *Metallosphaera sedula* | Tneu_0858 (arCOG05692) from *Thermoproteus neutrophilus* | E-value=3e-05 | Hit identified after second iteration.  Parameters: NR database (archaeal subset only); inclusion threshold E-value=1e-04; no low complexity filtering; no composition-based statistics |

Results of sequence similarity searches for highly diverged archaeal RecJ homologs
