# Supplementary material for: The CMG (CDC45/RecJ, MCM, GINS) complex is a conserved component of the DNA replication system in all archaea and eukaryotes
Source: Biol Direct. 2012 Feb 13;7:7. doi: 10.1186/1745-6150-7-7 (PMC3307487; doi:10.1186/1745-6150-7-7)
Supplement: Additional file 5 — The multiple alignment and secondary structure prediction for arCOG05692 family. Multiple alignment, secondary structure prediction and map of subdomains for predicted RecJ homologs of arCOG05692. [file 1745-6150-7-7-S5.DOCX]

**Multiple alignment and secondary structure prediction for arCOG05692 family**

The MUSCLE program [[1](#_ENREF_1)] was used for construction of the multiple sequence alignment. The sequences are denoted by their GI numbers and complete species name. The Jpred [[2](#_ENREF_2)] secondary structure prediction is shown underneath the multiple alignment; “H” indicates positions for α-helices and “E” - β-strands. The putative distinct structural regions are shown as follows: regions Cyan – DHH catalytic inactivated domain; green – DHH alpha helical region; magenta – potential connector helix; yellow – DHHA1 domain. For DHHA1 domain alignment with the corresponding domain of Sh1221 protein from Staphylococcus haemolyticus (PDB: 3dev), COG0618, is shown.

327310280 Thermoproteus_uzoniensis_768-20 -MELEQFFLNNINKILEYLDQDILTIYIRKTVDALVAGYFLMNKFADTAQLRLADWPPESGICLGFRCGGFYMYENEVGVEGSSIKL

126459787 Pyrobaculum_calidifontis_JCM_11548 MESFKNFIIDHIDRVLEYLKQNPLTIFVRRSVDAYMAAYALAAALGETTHVAVADWPPQRGVCVGFRCDGFYIAEGEAGVDDAKLAV

171185322 Thermoproteus_neutrophilus_V24Sta -MDLKNFIIDNVDRFLEYLKQNPLVIYARRSVDAYLAAYALAAALGETAQVSVVDWPPQAGVCVGFRCEGMYITEWGVGVDDRRFDA

119872291 Pyrobaculum_islandicum_DSM_4184 -MDFKNFVIDHIDRVLEYLKQNPLIIYTRRSVDAYLATYALIEAFGETAQLSVVDWPPKSGICIGFKCEGMYITEREVGIDNDRYNV

145592170 Pyrobaculum_arsenaticum_DSM_13514 -MDVRNFIIENIVRLLEYLKQNPLYVYTRRSVDAYLAAYALVASMGETAQVAVADWPPRAGICIGFKCDGFYITERGVGIDDKFHAS

18314102 Pyrobaculum_aerophilum_str-_IM2 -MDIKNFIIDNIDRILEYLKQNPLYIYARRSVDAYLAAYSLAASLGETAQVAVVDWPPQVGICIGFKCEGLYITERGIGLDDKVYTS

Jnet 18314102 -----HHHHHHHHHHHHHHH---EEEEEEH-HHHHHHHHHHHHH-----EEEEE-------EEEEEEE--EEEE-------------

3devA structure ---------------------------------------------------------------------------------------

3devA ---------------------------------------------------------------------------------------

327310280 Thermoproteus_uzoniensis_768-20 PTPLPISYIIYKLARSIISLNKDDILELYLGIFSWLVDNCTLRCEDPVELVGELETRRGFSLPFPDK-PLGEALSLLTLPLLPGVLG

126459787 Pyrobaculum_calidifontis_JCM_11548 -EPTSLSHMVSLIILSLSPLERHVHKALYVGHYSWSVDVCERNCQVPKELAVG-DERLAVVFPGLKR-GVKRALSLSTLPIVPGVFG

171185322 Thermoproteus_neutrophilus_V24Sta -GFTSMSHLASVLIQSLSPLEEQIHRSLYAGHYSWSVDYCEYGCPPPREILKG-DERLAVAFPFLEKLPARRALSLSTLPIVPGVTG

119872291 Pyrobaculum_islandicum_DSM_4184 -EFTSISHLVAIIIQSLSPLEEHIHRALYAGHYSWSVDYCEYKCPIPKELLKG-DEKLAIVFPFLDTLPVEKALSLSTLPILPGITG

145592170 Pyrobaculum_arsenaticum_DSM_13514 -GFTSISSLVASIITSISPLDEGVHKALYIGHYSWSVDYCDYKCPLPPELARG-DEQFAVVFPLVEEYPLGKALSLSTLPLIPGVTG

18314102 Pyrobaculum_aerophilum_str-_IM2 -DFTSMSHIVAHIITSLSPLEEDVYKALFIGHYSWSVDYCEYKCPPPRELNRG-DEKLAIVFPFVGELPANKALPLSTLPIIPGVTG

Jnet 18314102 -----HHHHHHHHHHH----HHHHHHHHEE------EEEE-------HHHH-----EEEEEE-------------------------

3devA structure ---------------------------------------------------------------------------------------

3devA ---------------------------------------------------------------------------------------

327310280 Thermoproteus_uzoniensis_768-20 RGIDEDKPLRALDQRRMLDVLDEALGRVYEAGFYPAIADKGLRYIPTEIEPTGRIIELEALLAGFTPDSQGVISYVDNLTKILDEIT

126459787 Pyrobaculum_calidifontis_JCM_11548 KAVEEDKRLEVMTRDEAISLLDWALGAVAAEGFHTAVLDKAVRPYSPTLNPADVAQRIEADLAGFV--DKDVEDYALNLAEAFYNVV

171185322 Thermoproteus_neutrophilus_V24Sta KPAEDSKLASAMGPDEALALLDWALGVVHSEGFHTAVLDKALRLYSPAFAPADFAARVEADLAGFV--GRDVELYVQNLADAFYGVV

119872291 Pyrobaculum_islandicum_DSM_4184 RGAENSKSIASMSSEEAVSLLDRALGVVYNEGFHTAVFDKAIKPYSFTYKPADIAIRIEADLAGFI--NRDISVYVSNLAETFYLLL

145592170 Pyrobaculum_arsenaticum_DSM_13514 REFD----LASLPADF-LAVLDWALGVVASEGFHTAILDKAIRHHSPAIRAATLAEKLEADLAGFV--NRELETYVASLAESFYNVV

18314102 Pyrobaculum_aerophilum_str-_IM2 REFD------SVPEDE-IRLLDWALGAVASEGFHTAILDKAVRYYSPEIKAADVAERLEADLANFI--DKGVEVYVSNLAENFYMIL

Jnet 18314102 -------------HHH-HHHHHHHHHHHH-----HHHHHHHH----------HHHHHHHHHHHHHH--HHHHHHHHHHHHHHHHHHH

3devA structure -------------------------------------------------------------------------------HHHHHHHH

3devA -----------------------------------------------------------------------------KDPKLXPFQG

327310280 Thermoproteus_uzoniensis_768-20 KSYQNNIINISN-TYYIYK---------LMNYLPYFSKLKDI-------LILRADIGKGFVASLIAPLKESQRLRAIADRLNDI-QY

126459787 Pyrobaculum_calidifontis_JCM_11548 KRAREGVVTLRN-PFYLHK---------IAPYLSYFAKSSQW-------AALRYETPSGSVVAVVPPHGLKGRLKAVAPLFAEVGQV

171185322 Thermoproteus_neutrophilus_V24Sta KRGREGVVPMQN-PFYVLK---------IPPYLSYYLRLSGW-------AALRYDAGRGHVVALVPPRGERDRLAKAAELLGEVGQV

119872291 Pyrobaculum_islandicum_DSM_4184 KKIKDSVITIQN-PFYIYK---------IPPYLSYYMKLTEW-------IILRYETTRGFILAVIPPMNKRDMLKNFANVLSEIGQT

145592170 Pyrobaculum_arsenaticum_DSM_13514 KRVKDGVVPLPN-PFYVYK---------LPPYLSYYLKLSNW-------VALRYETTRGYIAALVPPHGQNARLRKIAEELSEIGQV

18314102 Pyrobaculum_aerophilum_str-_IM2 KKVKEDVIPVQN-PFYIYK---------IPPYLSYYMKLANY-------VALKYDAPRGYVIALIPPFGEKAQLKTVASALAELGQV

Jnet 18314102 HHHH-----------EEEE-----------HHHHHHHHHHHH-------EEEEE----EEEEEEE-----HHHHHHHHHHHHHHHHH

3devA structure HHHH---------EEEEEE-Hhhhhh----HHHH---HHH--------EEEEEEE-----EEEEEEE-----HHHHHH---EEE---

3devA YVLQNFELsdSHEYCQIKITNdvlkqfdiqPNEASQFVNTVADIsgLKIWXFGVDe-gdqIRCRIRSKGITINDVANQFGGGGH---

327310280 Thermoproteus_uzoniensis_768-20 IAYDSSILVFVPRDRWPEVVELIK---------

126459787 Pyrobaculum_calidifontis_JCM_11548 FEFPTHLLLYVEAGRWGDFLKAYEKAKE-----

171185322 Thermoproteus_neutrophilus_V24Sta LRFPTHLVAYVERDKYADFLKIYEKGFV-----

119872291 Pyrobaculum_islandicum_DSM_4184 LVFPTHFIAYIESDKYADFLKIYEEVNK-----

145592170 Pyrobaculum_arsenaticum_DSM_13514 LEFPTHVIVYIEVGRYADFVRGFERLNEE----

18314102 Pyrobaculum_aerophilum_str-_IM2 LEFPTHIVAYIESNKYTDFLREYERIKK-----

Jnet 18314102 HH---EEEEEEE---HHHHHHHHHHH-------

3devA structure ------EEEEEE--HHHHHHHHHHHHHH-----

3devA ----PNASGVSVySWDEFEELAQALRQKLLEHH

1. Edgar RC: **MUSCLE: multiple sequence alignment with high accuracy and high throughput**. *Nucleic Acids Res* 2004, **32**(5):1792-1797.

2. Cuff JA, Clamp ME, Siddiqui AS, Finlay M, Barton GJ: **JPred: a consensus secondary structure prediction server**. *Bioinformatics* 1998, **14**(10):892-893.
